# Global Expansion of Jeffrey’s Insights: Jeffrey Modell Foundation’s Genetic Sequencing Program for Primary Immunodeficiency

**DOI:** 10.3389/fimmu.2022.906540

**Published:** 2022-06-10

**Authors:** Jessica Quinn, Vicki Modell, Britt Johnson, Sarah Poll, Swaroop Aradhya, Jordan S. Orange, Fred Modell

**Affiliations:** ^1^Jeffrey Modell Foundation, New York, NY, United States; ^2^Invitae, San Francisco, CA, United States

**Keywords:** genetic sequencing, sequencing, Next Generation Sequencing (NGS), Primary Immunodeficiency (PI), Jeffrey Modell Foundation (JMF), Jeffrey Modell Centers Network (JMCN), Inborn Errors of Immunity (IEI)

## Abstract

Genetic disorders that impair the immune system, known as Primary Immunodeficiencies (PI), include over 450 single-gene inborn errors of immunity. Timely and appropriate diagnosis and treatment is vital to quality of life (QOL) and sometimes survival, as patients are susceptible to frequent, persistent, severe, and sometimes life-threatening infections or autoimmunity. Suspected PI patients that do not have a genetic diagnosis often endure a prolonged, onerous, inefficient, and expensive experience, known as a diagnostic odyssey. The resulting diagnostic delay prohibits proper disease management and treatment, causing unnecessary distress and diminished QOL. Next-generation sequencing (NGS) offers relief from the distress of the diagnostic odyssey, but because of cost and barriers to access, it is regularly unobtainable. The Jeffrey Modell Foundation (JMF) introduced “Jeffrey’s Insights”, a no-charge genetic sequencing pilot program, in January 2019 for patients within the Jeffrey Modell Centers Network (JMCN) with an underlying PI, but no genetic diagnosis. Building on the success of the pilot program, JMF expanded it globally to more than 400 Centers in the JMCN in early 2020. The most current version of Invitae’s PI Panel available was used for this program. All participating clinicians were invited to complete a brief questionnaire assessing prior impediments to access and post-sequencing alterations in disease management and treatment. A total of 1,398 patients were tested, with 20.3% receiving a molecular diagnosis and many more receiving helpful diagnostic leads. Results obtained from genetic sequencing led to an alteration of clinical diagnosis, disease management, treatment, and genetic counseling in 39%, 38%, 35%, and 53% of patients, respectively. The global expansion of this program further underscores the crucial need for NGS for PI, along with its efficiency and potential cost savings. The results of this program to date further define rationale for the availability of comprehensive diagnostic NGS for patients with PI when requisitioned by an expert immunologist.

## Introduction

Genetic disorders that impair the immune system, known as Primary Immunodeficiencies (PI), include over 450 single-gene inborn errors of immunity (IEI) (1–4). Timely and appropriate diagnosis and treatment is vital to a patient’s quality of life (QOL) and sometimes their survival, as they are susceptible to frequent, persistent, severe, and sometimes life-threatening infections or autoimmunity (1, 5–8). Up to 1% of the population may have a PI; more than previous estimates (2, 9, 10).

Understanding of the immune system has improved markedly over the years, as has QOL for PI patients, in concert with advancement in next-generation sequencing (NGS) including multi-gene panels and whole-exome sequencing (ES), molecular diagnosis, and novel treatments (4, 11–15). Still, there is an urgent need for timely and enhanced management of these conditions (16, 17). Suspected PI patients that do not have a genetic diagnosis often endure frequent, costly, and unhelpful specialist visits and testing, known as a diagnostic odyssey (18). The resulting diagnostic delay has the potential to interfere with proper disease management and treatment, causing unnecessary distress, diminished QOL, and sometimes death. The diagnostic odyssey also can result in a substantial expense not only to the patients, but also to the health care system.

In many cases, NGS can offer the end of a diagnostic odyssey, through cost-efficient DNA sequencing. Available NGS panels cover a majority of genes causing PI and facilitate precision diagnosis, as various PIs present with overlapping clinical presentations. Furthermore, NGS has been demonstrated to influence or change patient diagnosis and disease management. A systematic review of studies utilizing NGS for PI found the median molecular diagnostic rate to be 25%, with a range of 15-46% (19). In one study, clinical diagnosis and clinical management were altered in 55% and 25% of patients with sequencing findings (11). Based upon this and other recent data for many individuals with a suspected PI, NGS is a crucial component of their clinical care. Importantly, early application not only maximizes benefits to the patient through potentially ending their diagnostic odyssey, but also provides increased value to health care systems.

Burdensome barriers such as cost, insurance, access, and interpretation, however, interfere with obtaining these beneficial resources in many cases (20). It has been suggested that insurers deny genetic testing more frequently than any other clinical test, with many requiring a geneticist referral, regardless of the expertise or qualifications of the ordering immunologist to determine the need for testing (20). These barriers result in suspected PI patients that have not been evaluated for genetic etiologies and remain within a diagnostic odyssey and risk excessive expense and anguish in pursuit of a diagnosis.

## Methods

### Jeffrey Modell Centers Network

For more than two decades, the Jeffrey Modell Centers Network (JMCN), a global network of specialized centers founded and established by the Jeffrey Modell Foundation (JMF), has provided a platform needed to optimize research advances, diagnosis, treatments, and connectivity. The network is constantly expanding, but currently includes 915 expert physicians, at 402 institutions, in 318 cities, and 87 countries across six continents. About a third, or 120, of the centers in the network are in the US, with 282 OUS. A list of expert physicians in the JMCN is shared, with permission, on JMF’s website through the “Find an Expert” tool, which can be accessed at http://info4pi.org/information-booth/find-an-expert. More than 258,000 patients are followed in the network, over 50% of which do not have a genetic diagnosis (21).

By leveraging the expertise of the JMCN, the unique advantage of high pre-test probability for a genetic diagnosis of PI for a given patient can be achieved. The JMCN offers direct coordinated access to expert clinicians who support a large number of individuals with a clinical diagnosis, but not yet having genetic confirmation, allowing us to connect those patients to valuable genetic diagnostics. We demonstrate, through the utilization of the JMCN, that an expert immunologist’s clinical index of suspicion alone should warrant application of NGS for PI diagnosis.

### The Pilot

JMF initiated a pilot program to provide clinical immunologists no-charge genetic sequencing to apply to individuals with a clinically diagnosed underlying PI in January 2019 (22). The program aimed to detect a genetic cause of disease, providing a molecular diagnosis to optimize management and treatment. Utilizing the unique JMCN, we hypothesized that immunologists having a high pre-test probability would thereby identify those patients who most needed tests, and through this, aimed to establish the value and utility of NGS for PI, essentially by putting the right tools in the right hands.

We invited 21 JMCN sites in 10 countries to participate. One hundred fifty-eight patients were tested, and 28 (21%) received a molecular diagnosis. It was reported by those in the pilot that cost was a major barrier in seeking genetic testing for these patients. Clinical diagnosis, disease management, treatment, and genetic counseling were altered in a substantial number of patients due to the genetic sequencing results obtained, in many cases even in the absence of receiving a molecular diagnosis. Importantly, there was a change in outcomes and an applicable therapy for nearly half and nearly all of the diagnosed patients, respectively (22).

### The Global Program

In early 2020, building on the success of the pilot program, JMF expanded access to the genetic sequencing program, “Jeffrey’s Insights”, globally to all centers in the JMCN. The program is still being offered with no charge to patients, physicians, hospitals, insurance companies, or government agencies. Through the expansion of this program, which is still ongoing, we seek to further establish the value and clinical efficiency of NGS for PI, utilizing the distinctive JMCN and the associated high pre-test probability. Additional goals include establishing the clinical diagnosis alteration rate and change in disease management due to the genetic sequencing results, outcomes facilitated *via* the JMCN and established connections with its expert immunologists.

Considering the outcomes of the pilot and the advantage of harnessing the JMCN’s experts’ ability to determine the most suitable patients, we anticipated that the molecular diagnostic rate from the global expansion of this program would remain in the range of 15-46%, as previously reported (19). We posit that the high clinical index of suspicion from an expert immunologist translates to high pre-test probability and will result in high diagnosis rates without additional tests, requirements, or justifications. Based upon the pilot, we predicted the rate of disease management alteration would be 25% and that revision of diagnosis would also continue to occur at a meaningful rate.

Modification of diagnosis and management has not only potential for improved patient outcomes but can provide considerable short- and long-term cost savings. We sought to also estimate the value and efficiency of NGS for PI from a health services standpoint, provide reasoning behind the need for extensive NGS diagnostics for PI, and provide support for increased access to NGS when warranted. We hypothesized that this program will help classify NGS as the primary intervention when a high clinical index of suspicion of a genetic PI by an expert immunologist exists.

In January 2020, the entire JMCN, approximately 400 sites, were invited to participate in the program’s global expansion and include their highest pre-test probability patients. We relied on the judgment of the expert immunologists as to which patients to include, with no specific criteria or additional justification applied or needed. For tests ordered by a participating clinician in the JMCN, one or more of the following clinical indications was suggested: (i) confirmed clinical diagnosis of PI without established genetic diagnosis, (ii) newborn screen suggestive of PI without established genetic diagnosis, (iii) suspected clinical diagnosis of PI, as per JMF’s 10 Warning Signs of PI (http://www.info4pi.org/library/educational-materials/10-warning-signs). Patient eligibility criteria were consistent with that previously described for the pilot program (22).

JMF partnered with Invitae to perform clinical NGS testing. Invitae is a fully certified clinical diagnostic laboratory which performs full-gene sequencing and intragenic deletion-duplication analysis using NGS technology. Invitae’s Primary Immunodeficiency Panel was used for this program, which included 207 genes until September 2020, when it was expanded to include 407 genes. A list of the genes included on each of these panels can be found in **Supplementary Tables 2, 3**. A detailed description of testing services has been previously described (22, 23). Variant interpretation was conducted in adherence with an expansion of the American College of Medical Genetics guidelines (24). Results were presented by the following variant classifications: pathogenic or likely pathogenic (P/LP) variants identified, uncertain (variants of uncertain significance -VUS- identified), and negative (no reportable variants identified). Increased risk alleles (common variants associated with an elevated risk of a disorder, but not a diagnosis) are widespread in the general population and so were excluded from the analysis. Results with pathogenic and likely pathogenic variants were further classified by their clinical relevance (i.e. carrier status, molecular diagnosis, see **Table 1**). Confirmed and likely molecular diagnoses were grouped together and are referred to as a “molecular diagnosis”.

**Table 1:** Clinical relevance of genetic test results.

Confirmed or Likely molecular diagnosis	Carrier status
1 heterozygous P/LP allele in AD gene	1 heterozygous allele in AR gene
1 hemizygous P/LP allele in XL gene in male	1 heterozygous allele in XL gene in female
2 heterozygous or 1 homozygous P/LP alleles in AR genes	
1 heterozygous P/LP allele and 1 VUS in AR genes	

Each column shows the possible genotypes identified in individuals with either a confirmed or likely molecular diagnosis or carrier result. For patients with heterozygous P/LP variants in genes that are associated with both AD and AR conditions, available information on the variant was used to determine if the P/LP variant was consistent with a molecular diagnosis of the AD condition or carrier status for the AR condition. AD, autosomal dominant inheritance; AR, autosomal recessive inheritance; P/LP, pathogenic or likely pathogenic variant; VUS, variant of uncertain significance; XL, X-linked inheritance.

Program protocol, including details related to specimen collection, consent, NGS testing, turn-around time, reporting, and Family Variant Testing, was consistent with that previously described for the pilot program (22). An updated questionnaire was distributed to all participating clinicians requesting de-identified information to assess access challenges and alterations in disease management and treatment for any individual tested (**Supplementary Figure 1**). This information was sought to better understand the value and ultimate impact of NGS testing for suspected PI patients.

## Results

### Geographic Reach

From January 2019 to August 2021, a total of 173 unique ordering clinicians have participated in this program, from 121 institutions in 98 cities within 45 countries. The global distribution can be seen in **Figure 1A**, and the distribution in the US can be seen in **Figure 1B**.

**Figure 1:**
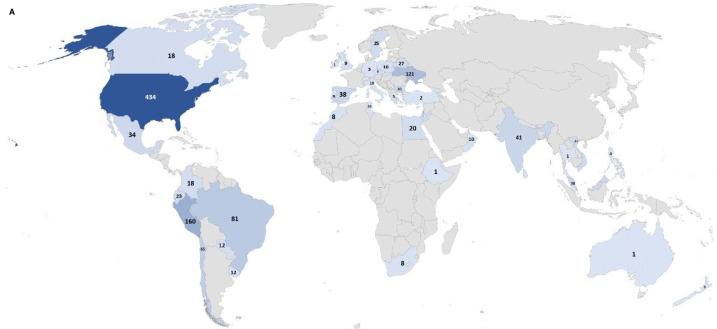
(Continued)

**Figure 1:**
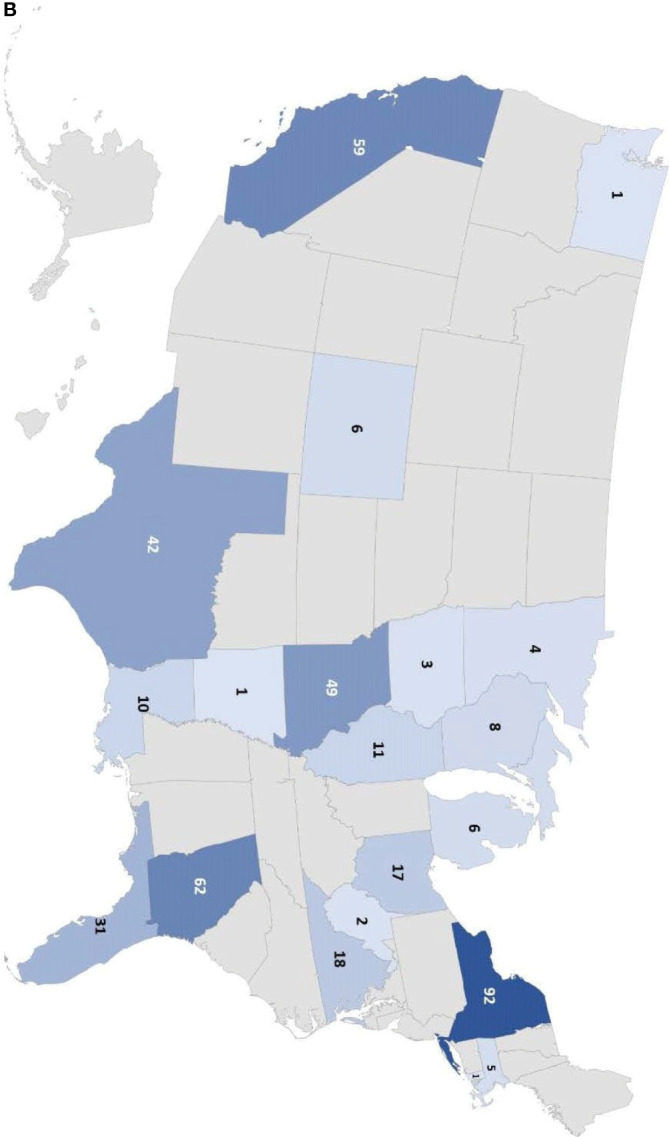
Number of individuals tested in the Jeffrey’s Insights program based on geographic region. **(A)** Number of individuals tested globally by country in the program. The numbers indicate the number of individuals tested from each country. Countries are colored from light to dark to correspond to the relative number of individuals tested from low to high, respectively. **(B)** Number of individuals tested in the U.S. by state. The numbers indicate the number of individuals tested from each state. The color scale represents the relative number of individuals tested in each state with the light blue corresponding to a few individuals tested to dark blue indicating the states with the highest numbers of individuals tested.

### Genetic Testing

A total of 1,398 unrelated patients and 27 family members have been tested through this program. Of these patients, 561 were tested using the 207 gene panel, while 838 were tested using the 407 gene panel after it was implemented in September 2020. There were 6,343 unique reportable variants (pathogenic, likely pathogenic, or VUS) identified, and 7,524 non-unique reportable variants identified, as some variants were identified in multiple patients. At least one reportable genetic variant was found in 1,373 patients (98.2%). A total of 455 unique P/LP variants and 667 non-unique P/LP variants were identified in 493 patients. Of the 667 non-unique P/LP variants identified, 68 (10.2%) were P/LP copy number variants (CNV). To note, 49 (72%) of these 68 P/LP CNVs were sub-genic and would be missed by traditional microarrays. **Table 2** shows all identified variants by the International Union of Immunological Societies (IUIS) category (2, 3).

**Table 2:** Number of non-unique variants identified by IUIS category.

IUIS category	P/LP positive	P/LP carrier	VUS	Total
Immunodeficiencies affecting cellular or humoral immunity (IUIS Table 1)	93	29	805	927
Combined immunodeficiencies associated with syndromic features (IUIS Table 2)	65	28	1231	1324
Antibody deficiencies (IUIS Table 3)	34	37	360	431
Disorders of immune dysregulation (IUIS Table 4)	66	26	787	879
Congenital defects of phagocyte number or function (IUIS Table 5)	45	28	598	671
Defects in Intrinsic and Innate Immunity (IUIS Table 6)	16	16	759	791
Autoinflammatory disorders (IUIS Table 7)	19	40	651	710
Complement deficiencies (IUIS Table 8)	12	21	266	299
Bone marrow failure (IUIS Table 9)	2	13	413	428
Multiple IUIS tables	23	0	124	147
Not in IUIS, but has overlapping symptoms	10	44	863	917
Total	385	282	6857	7524

The number of non-unique P/LP and VUS variants that were identified in patients tested in the Jeffrey’s Insights program. Variants were attributed to the different 2019 IUIS tables based on the gene that the variants were located in. P/LP variants were counted in the P/LP positive column if the variant was sufficient for a diagnosis (seen in a gene with autosomal dominant inheritance or X-linked inheritance in a male) or if it was seen with a second P/LP/VUS variant in a gene with autosomal recessive inheritance. P/LP variants were counted in the P/LP carrier column when a single P/LP variant and no other variant was identified in an autosomal recessive gene. IUIS, International Union of Immunological Societies; P/LP, pathogenic or likely pathogenic; VUS, variant of uncertain significance.

**Figure 2:**
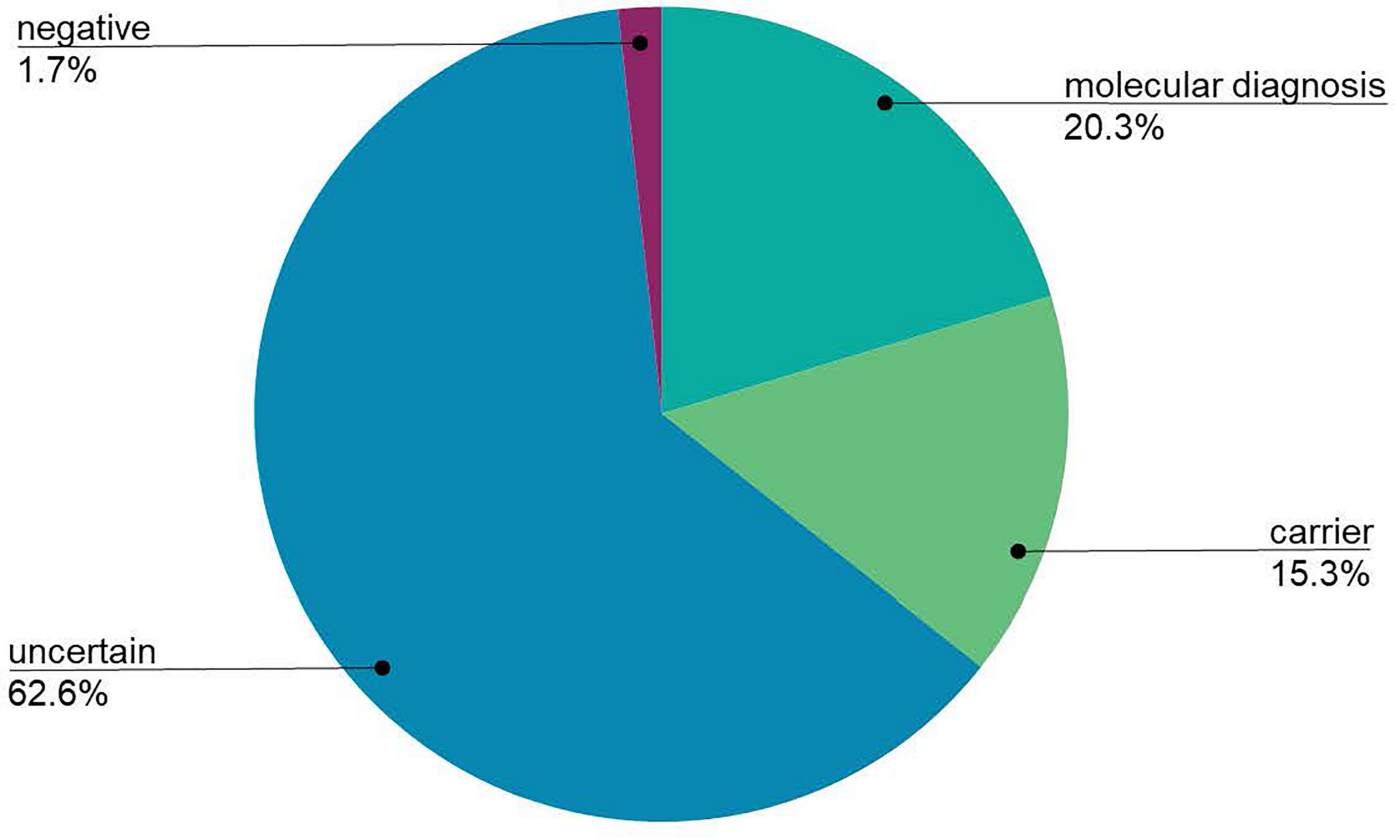
The global diagnostic yield of the Jeffrey’s Insights Program. To determine the overall diagnostic yield, individuals that received a confirmed molecular diagnosis or likely molecular diagnosis were grouped together (labeled molecular diagnosis in the figure). Individuals with 1 P/LP and 1 VUS in an autosomal recessive gene were considered to have a likely diagnosis. Uncertain results were patients who only received variants of uncertain significance. Individuals who did not have any pathogenic, likely pathogenic, or variants of uncertain significance identified were grouped in the negative results. Individuals who were tested for an indication of common variable immunodeficiency (CVID) were excluded from this calculation due to the primarily multifactorial nature of this condition.

**Figure 3:**
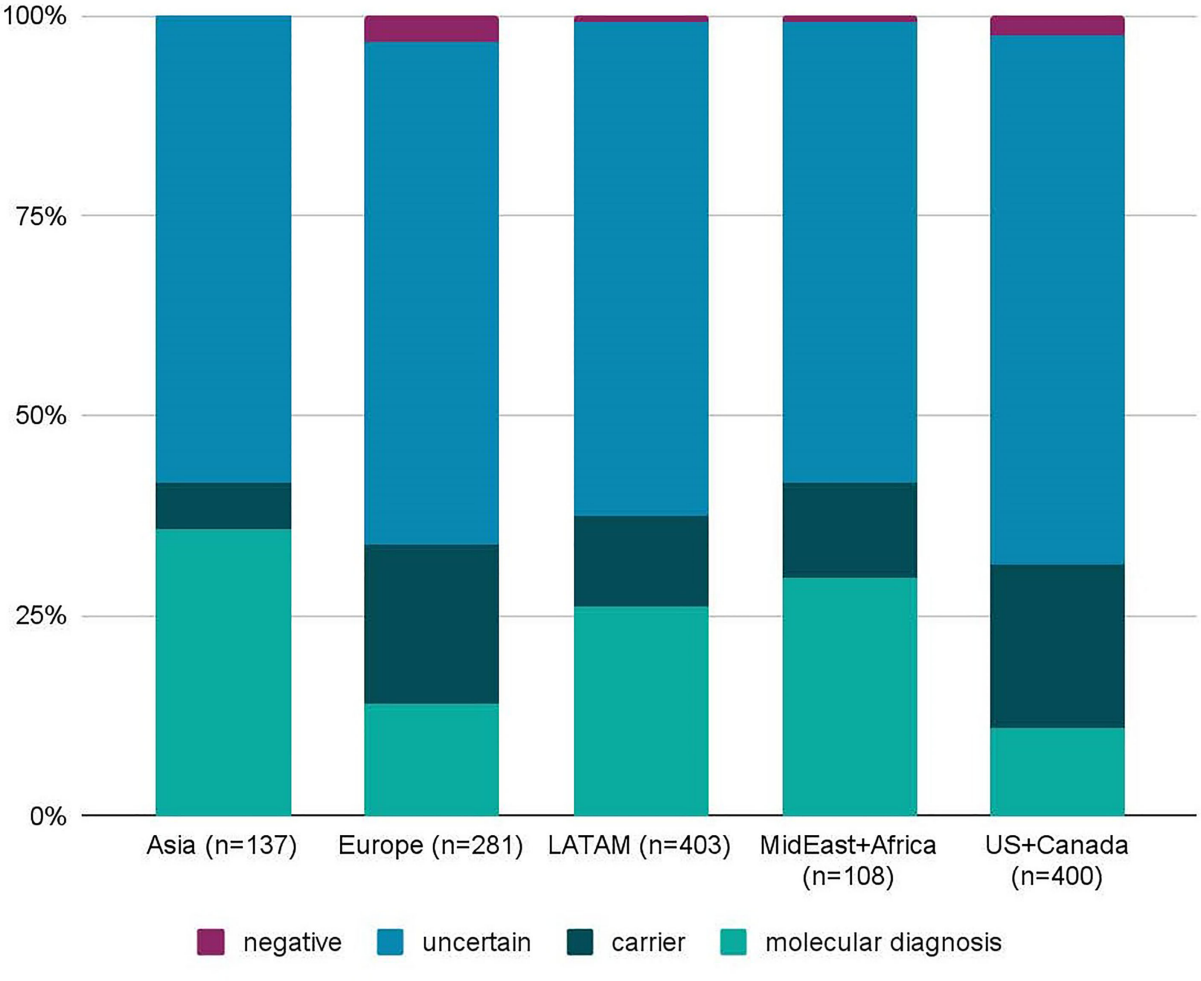
Diagnostic yield of the Jeffrey’s Insights program by geographic region. The diagnostic yield was calculated for patients from Asia, Europe, Latin America (LATAM), the Middle East (MidEast) plus Africa, and the United States (US) and Canada. Regional diagnostic yields were calculated the same way as the global diagnostic yield. Two out of 4 patients from Australia and New Zealand received a molecular diagnosis. However, these were excluded from the graph due to the low number of samples. n=number of samples.

Despite broad indications for genetic testing, 65 patients were suspected to have or were diagnosed with common variable immunodeficiency (CVID), which is usually multifactorial and less frequently due to a monogenic cause (25). Only four patients suspected to have CVID received a molecular diagnosis (CTLA4, SH2D1A, NFKB1, LRBA), and one patient was identified as a heterozygous carrier of a TACI pathogenic mutation, which is not diagnostic alone, but is considered a risk factor for CVID. A total of 1,333 patients received genetic testing for suspected monogenic disorders, excluding the CVID patients, and 271 (20.3%) received a molecular diagnosis (**Figure 2**). Additionally, 204 patients (15.3%) were found to be carriers of AR or XL conditions. Importantly, four patients had dual diagnoses with glucose-6-phosphate dehydrogenase (G6PD) deficiency: X-linked SCID (*IL2RG*), C6 deficiency (*C6*), IPEX syndrome (*FOXP3*), DiGeorge syndrome (*TBX1*). The diagnostic yield was not significantly different between individuals tested on the 207 gene panel (19.6%) and individuals tested on the 407 gene panel (20.8%; χ2 p-value = 0.3085). Geographically, diagnostic yield varied by region, with 35.8%, 29.6%, and 26.1% of patients receiving a molecular diagnosis in Asia, the Middle East or Africa, and Latin America, respectively (**Figure 3**). Europe had a diagnostic yield of 13.9% and the US and Canada had a combined diagnostic yield of 11%. Of the 27 family members that were tested, two received a molecular diagnosis and six received carrier results. **Supplementary Table 1** displays the molecular diagnoses attributed to known pathogenic variants categorized by gene per the IUIS Expert Committee classification of IEI.

The age of all individuals tested ranged from 0-87 years with an average of 12.2 years. Of those receiving a molecular diagnosis, 81% were tested during the pediatric period, with an average age of 8.7 years. Children aged 0-5 years had the highest diagnostic yield, with a 25% molecular diagnostic rate. In those 6-17 years old, 17% received a molecular diagnosis compared to 13% in patients 18 and older. This data is displayed in **Table 3**.

**Table 3:** Cohort demographics of the Jeffrey’s Insights Program.

	Number of patients (%)	Positive patients (diagnostic rate, %)
**Gender**		
**Male**	816 (58)	168 (21)
**Female**	582 (42)	107 (18)
**Total**	1398	275
**Age (years)**		
**≤5**	596 (43)	151 (25)
**6-17**	533 (38)	90 (17)
**18+**	269 (19)	34 (13)
**Total**	1398	275

Gender and age demographics of the total cohort are shown in the second column both in absolute number of individuals as well as the percentage of the total cohort in parentheses. Gender and age demographics of the subset of individuals with confirmed or likely molecular diagnoses are shown in the third column. The parentheses indicate the diagnostic yield for each of the demographic categories.

### The JMF Questionnaire

Participating clinicians were invited to complete a questionnaire to acquire additional insight into patient care and access before NGS and alterations to disease management and treatment after NGS (**Supplementary Figure 1**). Questionnaires were received for a total of 708 (50.6%) of the 1,398 participating patients.

Participating physicians reported that prior to NGS testing, patients visited a health care provider 7.68 times on average in the previous 12 months. It was also reported that in the previous 12 months, 59%, 47%, and 18% of patients had been admitted to the hospital, visited the emergency room, and been admitted to the ICU at least once, respectively. Notably, there were some patients with multiple admissions. Of the participating patients, 14% reportedly did not seek care and 73% did not seek NGS testing due to cost. Access to NGS testing was reported as an obstacle for 67% of the patients. Before genetic sequencing, the average annual estimated cost of care was $115,138. Only approximately 15% of patients were reported to have insurance coverage for genetic testing.

**Figure 4:**
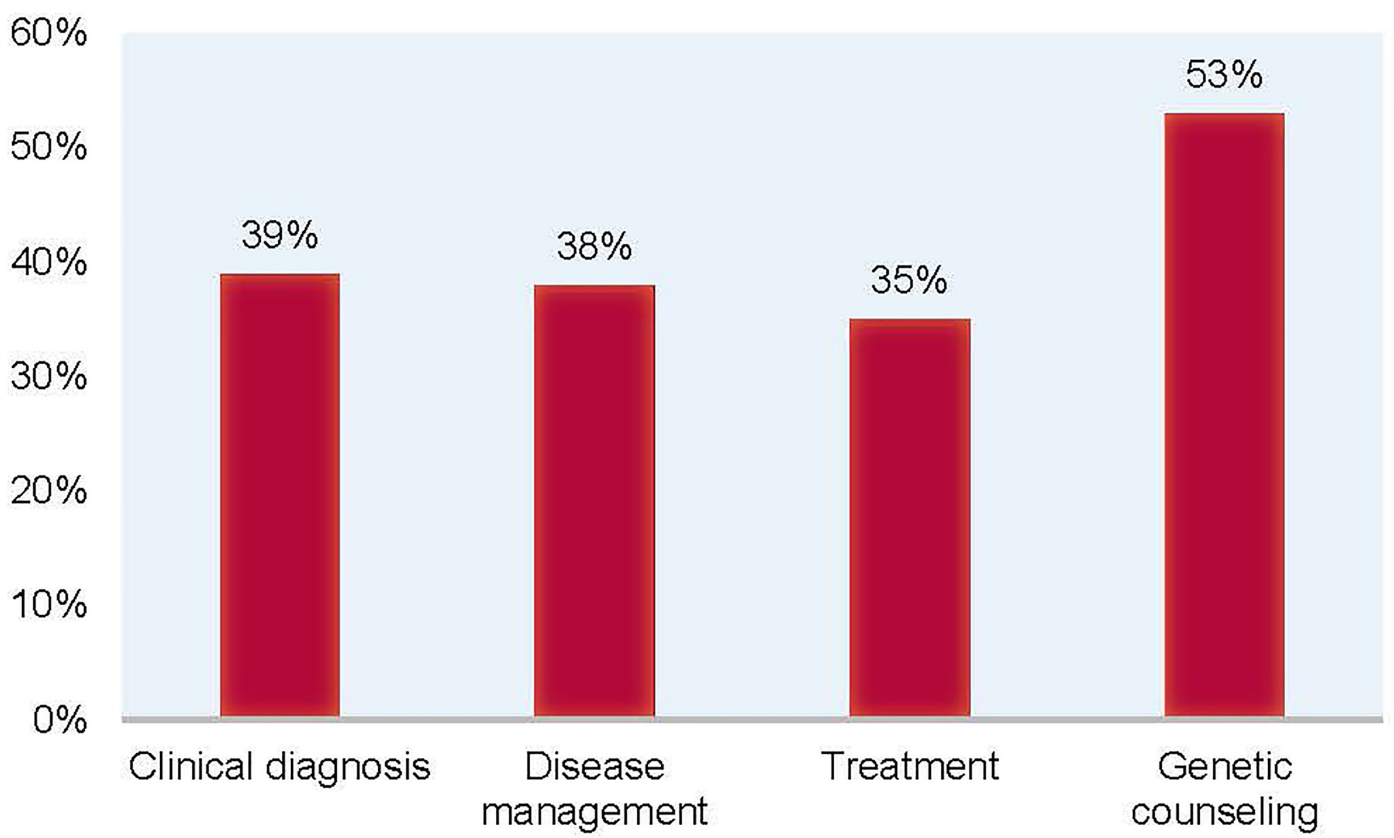
Alteration rates of clinical diagnosis, disease management, treatment, and genetic counseling in patients after NGS testing. Through the questionnaire, participating clinicians reported that based on the results of NGS testing, clinical diagnosis was altered in 39% of patients, disease management was altered in 38% of patients, treatment was altered in 35% of patients, and genetic counseling was altered in 53% of patients.

Clinicians reported having a suspicion of a specific diagnosis 75% of the time, but ended up altering their suspected diagnosis 39% of the time, based on the results of NGS testing. Overall, after NGS testing, whether or not a definitive molecular diagnosis was received, clinical diagnosis, disease management, treatment, and genetic counseling were altered in 39%, 38%, 35%, and 53% of patients, respectively (**Figure 4**). For 21% of participating patients, at-risk or affected family members were found. It was reported that there was a relevant therapy for 53% of the patients. Remarkably, 32% of patients reportedly had a change in outcomes based on the results of NGS testing.

### Costs of Care

Leveraging JMF’s previous economic proficiency in health care for PI (21, 26), estimated US dollar values were assigned to conditions that frequently affect patients with PI (**Table 4**) (27–31). As the JMF Questionnaire demonstrates, timely and accurate diagnosis, disease management, and treatment, made possible through NGS testing, could result in improved outcomes and a substantial reduction in the number of episodes of these conditions, as well as the associated costs.

**Table 4:** Costs of the most frequent conditions affecting patients with PI.

Condition	Average no. of episodes	Cost per episode	Annual cost
Persistent otitis media	4.2	$607	$2,549
Serious sinus & upper respiratory infections	4.6	$1,125	$5,175
Viral infections	3.7	$2,038	$7,540
Acute bronchitis	3.1	$468	$1,450
Bacterial pneumonias	2.8	$4,748	$13,294
Bronchiectasis	4.3	$2,136	$9,184
Hospitalization days	19.8	$2,607	$51,618
Physician/ER visits	70.8	$367	$25,983
Days on antibiotics	166.2	$5	$831
School/workdays missed	33.9	$200	$6,780
Total per patient			$124,404

The average number of episodes of each condition is listed in the second column. The estimated cost per episode and the associated total annual cost are listed in the third and fourth columns. The total annual cost per patient is shown in the bottom row of the fourth column. All costs are US based.

## Discussion

It is known that patients with a presumed PI, but no genetic diagnosis, often endure a long, onerous, inefficient, and expensive diagnostic odyssey that can delay or prevent accurate diagnosis, disease management, and treatment, leading to unnecessary distress and a diminished QOL. NGS offers a path to hope, understanding, and ultimately relief for these patients, but it is frequently inaccessible for a myriad of reasons, including insurance coverage and cost.

The JMCN is uniquely positioned to facilitate targeted access to expert immunologists who care for a larger number of suspected PI patients that have no genetic diagnosis, and has allowed us to demonstrate, through the global expansion of this program, the importance, efficiency, and crucial need of NGS testing for PI, which we argue, should be considered a first tier test used early in any patient that is suspected to have a PI. This program also highlights that expert clinicians are highly qualified to determine which patients need NGS. Restrictive eligibility criteria were intentionally not applied, as we believed in the value of an expert immunologist’s expertise and their likely high pretest probability in identifying the right patients for testing.

To date, the global expansion of this program has resulted in a diagnostic yield of 20.3%, which falls in the predicted 15-46% range described in a recent systematic review (19). The systematic review observed a 25% median diagnostic rate across all eight studies included, with four ranging from 15-25% and four ranging from 40-46%. Notably, of the four studies reporting diagnostic rates in the 40-46% range, one used ES (11), one used a test panel with a greater number of genes (32), one tested a highly consanguineous population (33), and one tested patients with at least one known causal mutation (34). Additionally, Invitae previously shared a 7% internal diagnostic rate for their 207 gene PI panel at the 2018 American Society of Human Genetics meeting (35). We posit that high pre-test probability attained by utilizing expert immunologists resulted in the higher diagnostic rate of 20.3%, illustrating the strength of their judgment and capability in recognizing those patients most in need of NGS testing.

When looking at diagnostic yield by region, we saw the highest diagnostic yield for patients from Asia (35.8%), the Middle East or Africa (29.6%), and LATAM (26.1%), highlighting that NGS testing for PI is an important diagnostic tool for diverse populations of different ethnic backgrounds and should be considered as a first tier test early in patients with a suspicion for PI worldwide.

A positive molecular diagnosis in a patient can often trigger cascade testing in relatives who may be at risk for the condition, who want to know their carrier status for future family planning, or who need to know their genetic status while being screened as a potential donor for their affected family member. Although this program was not broadly utilized for cascade testing (n=27), two relatives received a molecular diagnosis and six received carrier status results. This information may allow them to have access to treatments or proactive clinical management, or inform family planning and donor selection decisions.

In the global expansion of this program, 20.3% of patients received a molecular diagnosis. Clinical diagnosis, disease management, treatment, and genetic counseling were altered in 39%, 38%, 35%, and 53% of patients, respectively, as reported for 50.6% of participating patients. This suggests that non-diagnostic results can still be used by clinicians in decisions related to patient medical management and emphasizes the value of NGS testing, even without the identification of a definitive genetic diagnosis. Additionally, the findings highlight the need for unrestricted NGS testing for PI early in the diagnostic journey when there is a high suspicion of disease by an expert immunologist.

Further investigation into the rates of alteration of clinical diagnosis, disease management, treatment, and genetic counseling in patients receiving a molecular diagnosis compared to those receiving non-diagnostic results could be very informative and beneficial as this program progresses. Moving forward, it would also be useful to include further analysis regarding gender and age by gene. Additionally, further investigation into the implications of misdiagnosis and inappropriate treatment specifically in patients participating in this program could be beneficial in the future.

The JMF Questionnaire revealed patients across the globe face obstacles when seeking NGS testing including inaccessibility, high costs, and insurance denials. Consequentially, the exorbitant costs, both monetary and personal, of numerous hospital, emergency room, and ICU admissions are a significant burden for these patients. Alteration of diagnosis and disease management based on results of NGS testing not only has the potential to result in better patient outcomes but is also estimated to lead to considerable cost-savings. This point is illustrated in **Table 4**, where we leveraged our economic insight into PI to assign estimated US dollar values to conditions that often affect patients with PI (21, 26–31). The potential expenses for a patient that is not properly diagnosed are exorbitant. Importantly, NGS testing has the potential to substantially lower these costs by providing an accurate diagnosis, leading to proper disease management and treatment. We recognize that these costs are US based and will vary depending on the diagnosis and location of the patient, but assert that the potential cost-savings extrapolated from this information remains substantial regardless of diagnosis or geographic location. This cost reduction does not even account for high-priced immunologic and phenotypic tests that are frequently utilized repeatedly in the absence of a conclusive genetic diagnosis. Notably, most of these additional high-priced tests are not subjected to the same access or insurance barriers as NGS testing and represent an actual economic burden to payors and health systems. Considering only the potential cost-savings of NGS testing for PI, there is sufficient rationale for comprehensive NGS diagnostics for PI when applied by expert immunologists, as well as justification for their increased access to NGS testing in most circumstances.

## Conclusion

Through the global expansion of this program and by taking advantage of the uniquely positioned JMCN, we were able to show the efficiency, impact, and value of NGS testing for PI by expert immunologists. There is a crucial need for this service, as most expert clinicians in the JMCN have suspected PI patients with restricted or no access to NGS testing, despite their adeptness at determining when it is warranted. Overall, we justify the need for extensive NGS testing for suspected PI patients when requested by an expert immunologist, as they offer an inherent high pre-test probability and should have unencumbered access in most circumstances. We show that NGS testing has a significant diagnostic yield for patients with suspected PI globally and can lead to changes in clinical diagnosis, disease management, treatment, and genetic counseling, even in the absence of a definitive diagnosis, and has the potential to save healthcare dollars when implemented early. Accordingly, we advocate for health agencies, third party payers, and governments to recognize NGS testing for PI as a first step in intervention when an expert immunologist has a high suspicion of a genetic etiology for a clinically diagnosed patient.

## Data Availability Statement

The original contributions presented in the study are included in the article/**Supplementary Material**. Further inquiries can be directed to the corresponding author.

## Author Contributions

VM, JO, and FM conceived the program. JQ provided essential program oversight. JQ, BJ, SP, and SA provided vital data analyses. JQ drafted the initial manuscript. BJ and JO contributed substantial additions and revisions to the manuscript. All authors provided meaningful manuscript revisions. All authors read and approved the final manuscript.

## Conflict of Interest

JO: Consultant to Grifols, CSL, Takeda, Teva, Sobi, Jansen, Editas; ADMA, Gigagen, and Edity Scientific Advisory Boards; author and editor in immunology for Up To Date receiving royalties; patent related to genetic testing held by Children’s Hospital of Philadelphia. SP, SA, and BJ are current salaried employees of Invitae, including stock benefits.

The remaining authors declare that the research was conducted in the absence of any commercial or financial relationships that could be construed as a potential conflict of interest.

## Publisher’s Note

All claims expressed in this article are solely those of the authors and do not necessarily represent those of their affiliated organizations, or those of the publisher, the editors and the reviewers. Any product that may be evaluated in this article, or claim that may be made by its manufacturer, is not guaranteed or endorsed by the publisher.

## Abbreviations

PI, Primary Immunodeficiency; IEI, Inborn Errors of Immunity; QOL, Quality of life; ES, Exome sequencing; NGS, Next Generation Sequencing; DNA, Deoxyribonucleic acid; JMF, Jeffrey Modell Foundation; JMCN, Jeffrey Modell Centers Network; US, United States; OUS, Outside of the United States; P/LP, Pathogenic or likely pathogenic; VUS, Variants of uncertain significance; AD, Autosomal dominant; AR, Autosomal recessive; XL, X-linked; CNV, Copy number variant; IUIS, International Union of Immunological Societies; CVID, Common variable immune deficiency; ICU, Intensive care unit; IVIG, Intravenous immune globulin.
