# Supplementary material for: Global Expansion of Jeffrey’s Insights: Jeffrey Modell Foundation’s Genetic Sequencing Program for Primary Immunodeficiency
Source: Front Immunol. 2022 Jun 10;13:906540. doi: 10.3389/fimmu.2022.906540 (PMC9226364; doi:10.3389/fimmu.2022.906540)
Supplement: Supplementary file 1 [file DataSheet_1.pdf]

Supplementary Material

Supplementary Table 1. Molecular diagnoses by gene. Positive gene results are organized by the 2019 IUIS categories and the total number of individuals with positive molecular diagnoses in each gene are indicated. Rows in bold indicate homozygous variants.

IUIS Category	IUIS Table	Gene	No. of Patients	Genotypes	Effect
Immunodeficiencies affecting cellular or humoral immunity	1	ADA	5	**NM_000022.2:c.1021_1022del**	**p.Arg341Glyfs*8**
				**NM_000022.2:c.218+1G>A**	**Splice donor**
				**NM_000022.2:c.320T>C**	**p.Leu107Pro**
				NM_000022.2:c.646G>A; NM_000022.2:c.302G>A	p.Gly216Arg; p.Arg101Gln
				NM_000022.2:c.704G>A; NM_000022.2:c.646G>A	p.Arg235Gln; p.Gly216Arg
		CD3D	2	**NM_000732.4:c.202C>T**	**p.Arg68***
		CD40LG	7	NM_000074.2:c.289-?_346+?del	Deletion (Exon 3)
				NM_000074.2:c.31C>T	p.Arg11*
				NM_000074.2:c.347-?_*975+?del	Deletion (Exons 4-5)
				NM_000074.2:c.418T>C	p.Trp140Arg
				NM_000074.2:c.474del	p.Lys159Asnfs*3
				NM_000074.2:c.761C>T	p.Thr254Met
		DCLRE1C	4	**NM_001033855.2:c.-422-?_246+?del**	**Deletion (Exons 1-3)**
				**NM_001033855.2:c.1299_1306dup**	**p.Cys436***
		DOCK2	2	**NM_004946.2:c.316dup**	**p.Tyr106Leufs*35**
				NM_004946.2:c.4786C>T; NM_004946.2:c.1348_1349del	p.Arg1596*; p.Met450Valfs*12

		DOCK8	4	NM_203447.3:c.54-?_1285+?del	Deletion (Exons 2-11)
				NM_203447.3:c.5161C>T; NM_203447.3:c.5481dup	p.Gln1721*; p.Arg1828*
				**NM_203447.3:c.54-?_404+?del**	**Deletion (Exons 2-4)**
				**NM_203447.3:c.828-?_1125+?del**	**Deletion (Exons 8-10)**
		IL2RG	10	NM_000206.2:c.116-2A>G	Splice acceptor
				NM_000206.2:c.216C>A	p.Cys72*
				NM_000206.2:c.328G>T	p.Glu110*
				NM_000206.2:c.713G>A	p.Ser238Asn
				NM_000206.2:c.758-2A>G	Splice acceptor
				NM_000206.2:c.854G>A	p.Arg285Gln
				NM_000206.2:c.855-1G>A	Splice acceptor
				NM_000206.2:c.865C>T	p.Arg289*
				NM_000206.2:c.982C>T	p.Arg328*
		IL7R	5	**NM_002185.3:c.353G>A**	**p.Cys118Tyr**
				NM_002185.3:c.662G>T; NM_002185.3:c.83-2A>T	p.Ser221Ile; Splice acceptor
				**NM_002185.3:c.83-2A>T**	**Splice acceptor**
		JAK3	2	**NM_000215.3:c.1142+1G>C**	**Splice donor**
				**NM_000215.3:c.1645C>T**	**p.Arg549***
		NHEJ1	1	**NM_024782.2:c.178-1G>A**	**Splice acceptor**
		RAG1	3	NM_000448.2:c.2275C>T; NM_000448.2:c.1228C>T	p.Arg759Cys; p.Arg410Trp
				**NM_000448.2:c.256_257del**	**p.Lys86Valfs*33**
				**NM_000448.2:c.2689C>T**	**p.Arg897***
				**NM_000536.3:c.475C>T**	**p.Arg159Cys**

		RAG2	2	**NM_000536.3:c.686G>A**	**p.Arg229Gln**
		RFX5	1	**NM_000449.3:c.367_368del**	**p.Leu124Cysfs*21**
		RFXANK	1	**NM_003721.3:c.383del**	**p.Leu128Profs*76**
		STK4	1	**NM_006282.3:c.349C>T**	**p.Arg117***
		ZAP70	2	**NM_001079.3:c.1518C>A**	**p.Tyr506***
				**NM_001079.3:c.261C>G**	**p.Tyr87***
Combined immunodeficiencies associated with syndromic features	2	ATM	8	**NM_000051.3:c.186-?_3284+?del**	**Deletion (Exons 4-22)**
				NM_000051.3:c.2921+1G>A;<br>NM_000051.3:c.3802del	Splice donor;<br>p.Val1268*
				**NM_000051.3:c.3894dup**	**p.Ala1299Cysfs*3**
				NM_000051.3:c.4019_4029del;<br>NM_000051.3:c.2817del	p.Leu1340Cysfs*10;p.Lys940Asnfs*9
				NM_000051.3:c.8584+2T>C;<br>NM_000051.3:c.8147T>C	Splice donor;<br>p.Val2716Ala
				**NM_000051.3:c.8851-?_*3591+?del**	**Deletion (Exons 62-63)**
				**NM_000051.3:c.8883dup**	**p.Met2962Hisfs*16**
		CHD7	1	NM_017780.3:c.4944_4945del	p.Tyr1649Leufs*3
		DNMT3B	1	NM_006892.3:c.2292G>T;<br>NM_006892.3:c.1838T>C	p.Arg764Ser;<br>p.Val613Ala
		EPG5	2	**NM_020964.2:c.2662A>T**	**p.Lys888***
				**NM_020964.2:c.5583C>A**	**p.Cys1861***
		ERCC6L2	2	**NM_020207.4:c.1963C>T**	**p.Arg655***
				NM_020207.4:c.3603_3607dup;<br>NM_020207.4:c.1963C>T	p.Pro1203Hisfs*10;<br>p.Arg655*
		FOXN1	1	NM_003593.2:c.1201_1216del	p.Pro401Alafs*144
		NBN	1	NM_002485.4:c.657_661del;<br>NM_002485.4:c.808_809del	p.Lys219Asnfs*16;<br>p.Val270Cysfs*2

		NFKBIA	1	NM_020529.2:c.95G>A	p.Ser32Asn
		PNP	2	**NM_000270.3:c.244C>T**	**p.Gln82***
				**NM_000270.3:c.547dup**	**p.Glu183Glyfs*4**
		SP110	2	NM_004509.3:c.1116_1119del; NM_004509.3:c.1448-?_1590+?del	p.Arg373Serfs*22; Deletion (Exon 14)
				**NM_004509.3:c.686dup**	**p.Gln231Profs*5**
		TBX1	10	NM_080647.1:c.-129-?_*465+?del	Deletion (Entire coding sequence)
Antibody deficiencies	3	AICDA	1	**NM_020661.2:c.544-2A>G**	**Splice acceptor**
		BTK	13	c.216_220delins15	p.Pro73Leufs*15
				NM_000061.2:c.119A>G	p.Tyr40Cys
				NM_000061.2:c.1420dup	p.Thr474Asnfs*2
				NM_000061.2:c.1558C>T	p.Arg520*
				NM_000061.2:c.1574G>A	p.Arg525Gln
				NM_000061.2:c.1631+5G>C	Intronic
				NM_000061.2:c.1684C>T	p.Arg562Trp
				NM_000061.2:c.179_181del	p.Lys60del
				NM_000061.2:c.1901G>C	p.Trp634Ser
				NM_000061.2:c.1909-?_*438+?del	Deletion (Exon 19)
				NM_000061.2:c.1931T>C	p.Phe644Ser
				NM_000061.2:c.496C>T	p.Gln166*
				NM_000061.2:c.520+1G>A	Splice donor
		NFKB1	6	NM_003998.3:c.-467-?_*708+?del	Deletion (Entire coding sequence)
				NM_003998.3:c.2265T>A	p.Tyr755*
				NM_003998.3:c.2382T>G	p.Tyr794*

				NM_003998.3:c.357_358del	p.Cys119*
				NM_003998.3:c.836-1G>A	Splice acceptor
				NM_003998.3:c.879_880del	p.Val294Leufs*14
		NFKB2	1	NM_001077494.3:c.2611C>T	p.Gln871*
		PIK3CD	5	NM_005026.3:c.241G>A	p.Glu81Lys
				NM_005026.3:c.3061G>A	p.Glu1021Lys
		PIK3R1	4	NM_181523.2:c.1344dup	p.Leu449Ilefs*3
				NM_181523.2:c.1425+1G>A	Splice donor
				NM_181523.2:c.1425+1G>T	Splice donor
		TCF3	2	Alternate transcript NM_001136139.3:c.1663G>A	p.Glu555Lys
Disorders of immune dysregulation	4	AIRE	2	**NM_000383.3:c.560C>G**	**p.Ser187***
				NM_000383.3:c.967_979del; NM_000383.3:c.1045C>T	p.Leu323Serfs*51; p.Gln349*
		AP3B1	1	**NM_003664.4:c.779G>A**	**p.Trp260***
		CTLA4	2	c.38_43delinsCTGGCACA	p.Leu13Profs*60
				NM_005214.4:c.238C>T	p.Gln80*
		FAS	2	NM_000043.5:c.197-1G>A	Splice acceptor
				NM_000043.5:c.442A>T	p.Lys148*
		FOXP3	4	NM_014009.3:c.1099T>C	p.Phe367Leu
				NM_014009.3:c.1150G>A	p.Ala384Thr
				NM_014009.3:c.2T>A	p.Met1?
		LRBA	5	**NM_006726.4:c.3286_3287del**	**p.Phe1096Leufs*3**
				**NM_006726.4:c.5581-?_5645+?del**	**Deletion (Exon 35)**
				**NM_006726.4:c.5755-?_5921+?dup**	**Gain (Exon 37)**
				**NM_006726.4:c.7970T>G**	**p.Ile2657Ser**

				**NM_006726.4:c.893del**	**p.Lys298Serfs*7**
		LYST	4	**NM_000081.3:c.193-?_*1909+?del**	**Deletion (Exons 4-53)**
				NM_000081.3:c.2832del; NM_000081.3:c.1406T>A	p.Ser945Leufs*29; p.Leu469*
				**NM_000081.3:c.3713-?_4862+?dup**	**Gain (Exons 9-14)**
				NM_000081.3:c.6254-?_*1909+?del; NM_000081.3:c.9449del	Deletion (Exons 23-53); p.Asn3150Thrfs*35
		PEPD	1	**NM_000285.3:c.504-1G>A**	**Splice acceptor**
		PRF1	1	**NM_001083116.1:c.445G>A**	**p.Gly149Ser**
		RASGRP1	2	**NM_005739.3:c.1633C>T**	**p.Gln545***
				**NM_005739.3:c.2180G>A**	**p.Trp727***
		SH2D1A	4	NM_002351.4:c.-361-?_*1769+?del	Deletion (Entire coding sequence)
				NM_002351.4:c.158C>T	p.Thr53Ile
				NM_002351.4:c.163C>T	p.Arg55*
		STAT1	4	NM_007315.3:c.820C>T	p.Arg274Trp
				NM_007315.3:c.862A>G	p.Thr288Ala
		STXBP2	3	**NM_006949.3:c.1247-1G>C**	**Splice acceptor**
				**NM_006949.3:c.1621G>A**	**p.Gly541Ser**
		UNC13D	1	**NM_199242.2:c.766C>T**	**p.Arg256***
		XIAP	4	NM_001167.3:c.1141C>T	p.Arg381*
				NM_001167.3:c.664C>T	p.Arg222*
				NM_001167.3:c.894_898del	p.Lys299Leufs*9
				NM_001167.3:c.978-?_1099+?del	Deletion (Exons 4-5)

Congenital defects of phagocyte number or function	5	CTSC	1	**Complex rearrangement**	**Exons 1-3**
		CYBA	1	**NM_000101.3:c.385G>T**	**p.Glu129***
		CYBB	10	NM_000397.3:c.-61-?_*2544+?del	Deletion (Entire coding sequence)
				NM_000397.3:c.1012C>T	p.His338Tyr
				NM_000397.3:c.1519C>T	p.Gln507*
				NM_000397.3:c.252+5G>C	Intronic
				NM_000397.3:c.253-8A>G	Intronic
				NM_000397.3:c.469C>T	p.Arg157*
				NM_000397.3:c.565del	p.Ile189Leufs*25
				NM_000397.3:c.675-2A>G	Splice acceptor
				NM_000397.3:c.676C>T	p.Arg226*
				NM_000397.3:c.742dup	p.Ile248Asnfs*36
		ELANE	5	NM_001972.2:c.367-8C>A	Intronic
				NM_001972.2:c.377C>T	p.Ser126Leu
				NM_001972.2:c.597+1G>A	Splice donor
				NM_001972.2:c.607G>C	p.Gly203Arg
		G6PD	12	c.[202G>A;376A>G]	p.[Val68Met;Asn126Asp]
				c.[376A>G;968T>C]	p.[Asn126Asp;Leu323Pro]
				NM_001042351.2:c.1360C>T	p.Arg454Cys
				NM_001042351.2:c.392G>T	p.Gly131Val
				NM_001042351.2:c.563C>T	p.Ser188Phe
				NM_001042351.2:c.844G>C	p.Asp282His
				NM_001042351.2:c.871G>A	p.Val291Met
				NM_001042351.2:c.949G>A	p.Glu317Lys
				NM_032638.4:c.1019C>A	p.Ser340*

		GATA2	3	NM_032638.4:c.1081C>T	p.Arg361Cys
				NM_032638.4:c.437dup	p.Gly147Trpfs*38
		ITGB2	1	**NM_000211.4:c.562C>T**	**p.Arg188***
		NCF2	2	**NM_000433.3:c.669+1G>A**	**Splice donor**
				NM_000433.3:c.714-?_924+?dup; NM_000433.3:c.367-1G>A	Gain (Exons 8-9); Splice acceptor
		SRP54	2	NM_003136.3:c.349_351del	p.Thr117del
Defects in Intrinsic and Innate Immunity	6	CXCR4	3	NM_003467.2:c.1000C>T	p.Arg334*
		IFNGR1	2	NM_000416.2:c.781del	p.Ser262Alafs*15
				NM_000416.2:c.819_822del	p.Asn274Hisfs*2
		IL12B	1	**NM_002187.2:c.482+1G>T**	**Splice donor**
		IL12RB1	3	**NM_005535.2:c.1456C>T**	**p.Arg486***
				**NM_005535.2:c.1791+2T>G**	**Splice donor**
				**NM_005535.2:c.962C>A**	**p.Ser321***
		IRAK4	1	NM_016123.3:c.1135G>T; NM_016123.3:c.1188+520A>G	p.Glu379*; Intronic
Autoinflammatory disorders	7	ADA2	3	**NM_001282225.1:c.1085G>A**	**p.Trp362***
				**NM_001282225.1:c.1358A>G**	**p.Tyr453Cys**
				NM_001282225.1:c.973-2A>G; NM_001282225.1:c.934C>T	Splice acceptor; p.Arg312*
		CARD14	1	NM_024110.4:c.349G>A	p.Gly117Ser
		MVK	1	**NM_000431.3:c.829C>T**	**p.Arg277Cys**
		NLRP3	2	NM_004895.4:c.1219A>C	p.Thr407Pro
				NM_004895.4:c.784C>T	p.Arg262Trp
		NOD2	1	NM_022162.2:c.1538T>C	p.Met513Thr
		PSTPIP1	1	NM_003978.3:c.688G>A	p.Ala230Thr

		TMEM173	1	NM_198282.3:c.461A>G	p.Asn154Ser
		TNFAIP3	3	NM_006290.3:c.120del	p.Phe40Leufs*56
				NM_006290.3:c.1904_1905del	p.Lys635Thrfs*36
				NM_006290.3:c.547C>T	p.Arg183*
Complement deficiencies	8	C6	1	NM_000065.3:c.1138del; NM_000065.3:c.1879del	p.Gln380Serfs*7; p.Asp627Thrfs*4
		CFI	1	NM_000204.4:c.772G>A	p.Ala258Thr
		SERPING1	9	NM_000062.2:c.-191-?_550+?del	Deletion (Exons 1-3)
				NM_000062.2:c.1250-1G>C	Splice acceptor
				NM_000062.2:c.1361del	p.Val454Glyfs*122
				NM_000062.2:c.305_317del	p.Pro102Leufs*42
				NM_000062.2:c.550G>A	p.Gly184Arg
				NM_000062.2:c.685+1G>A	Splice donor
				NM_000062.2:c.686-?_1029+?del	Deletion (Exons 5-6)
Bone marrow failure	9	PARN	1	Partial Deletion	Exon 19
		RTEL1	1	NM_001283009.1:c.1266+3_1266+80del	Intronic
Multiple IUIS tables	1, 2, 3	CARD11	2	NM_032415.5:c.146G>A	p.Cys49Tyr
				NM_032415.5:c.368G>A	p.Gly123Asp
	1, 3, 5	RAC2	1	NM_002872.4:c.184G>A	p.Glu62Lys
	2, 4	STAT3	12	NM_139276.2:c.1144C>T	p.Arg382Trp
				NM_139276.2:c.1145G>A	p.Arg382Gln
				NM_139276.2:c.1387_1389del	p.Val463del
				NM_139276.2:c.1859C>G	p.Thr620Ser
				NM_139276.2:c.1910T>C	p.Val637Ala
				NM_139276.2:c.1976T>A	p.Ile659Asn

				NM_139276.2:c.2116C>A	p.Leu706Met
				NM_139276.2:c.861G>T	p.Leu287Phe
	2, 5	WAS	8	NM_000377.2:c.1085del	p.Pro362Glnfs*83
				NM_000377.2:c.121C>T	p.Arg41*
				NM_000377.2:c.1271dup	p.Leu425Profs*70
				NM_000377.2:c.1339-2A>G	Splice acceptor
				NM_000377.2:c.176del	p.Pro59Leufs*17
				NM_000377.2:c.723del	p.Ser242Valfs*19
				NM_000377.2:c.91G>A	p.Glu31Lys
Not in IUIS, but has overlapping symptoms	N/A	CBL	1	**NM_005188.3:c.1111T>C**	**p.Tyr371His**
		DUOX2	5	NM_014080.4:c.1153C>T	p.Gln385*
				**NM_014080.4:c.1461_1462delinsCA**	**p.Gly488Arg**
				NM_014080.4:c.1588A>T	p.Lys530*
				NM_014080.4:c.1606C>T	p.Arg536*
				Partial Deletion	Exon 23
		PMM2	1	NM_000303.2:c.422G>A; NM_000303.2:c.385G>A	p.Arg141His; p.Val129Met

Note: This table lists known pathogenic or likely pathogenic variants in adherence with the American College of Medical Genetics guidelines. Variants of unknown significance are not listed in this table and are not independently validated.

Supplementary Table 2. Genes on the 207 gene PI panel and their associated conditions

Gene	Condition(s)
ACD	AD/AR-DKC due to TPP1 deficiency
ACP5	Spondyloenchondro-dysplasia with immune dysregulation
ACTB	β-actin deficiency
ADA	Adenosine deaminase (ADA) deficiency
ADA2	ADA2 deficiency

ADAM17	ADAM17 deletion
ADAR	ADAR1 deficiency, Aicardi-Goutieres syndrome 6
AICDA	AID deficiency
AIRE	APECED (APS-1), autoimmune polyendocrinopathy with candidiasis and ectodermal dystrophy
AK2	Reticular dysgenesis, AK2 deficiency
AP3B1	Hermansky-Pudlak syndrome, type 2
ATM	Ataxia-telangiectasia
B2M	MHC class I deficiency
BCL10	BCL10 deficiency
BLNK	BLNK deficiency
BLOC1S6	Hermansky-Pudlak syndrome, type 9
BTK	BTK deficiency
CARD11	CARD11 deficiency, CARD11 gain of function
CARD14	CAMPS (CARD14 mediated psoriasis)
CARD9	CARD9 deficiency
CASP10	ALPS-Caspase 10
CASP8	ALPS IIb, caspase 8 deficiency
CD247	CD3ζ deficiency
CD27	CD27 deficiency
CD3D	CD3δ deficiency
CD3E	CD3ε deficiency
CD3G	CD3γ deficiency
CD40LG	CD40 ligand deficiency
CD79A	Igα deficiency
CD79B	Igβ deficiency
CD8A	CD8 deficiency
CEBPE	Specific granule deficiency
CHD7	CHARGE syndrome

CIITA	MHC class II deficiency
CLPB	3-Methylglutaconic aciduria
COPA	COPA defect, autoimmune interstitial lung, joint, and kidney disease (AILJK)
CORO1A	Coronin-1A deficiency
CR2	CD21 deficiency
CSF2RA	Pulmonary alveolar proteinosis
CSF3R	G-CSF receptor deficiency
CTC1	AR-DKC due to CTC1 deficiency
CTLA4	CTLA4 deficiency (ALPSV)
CTPS1	CTPS1 deficiency
CTSC	Papillon-Lefèvre Syndrome
CXCR4	WHIM (warts, hypogammaglo- bulinemia, infections, myelokathexis) syndrome
CYBA	Autosomal recessive CGD
CYBB	X-linked chronic granulomatous disease (CGD)
DCLRE1B	AR-DKC due to DCLRE1B deficiency
DCLRE1C	DCLRE1C (Artemis) deficiency
DKC1	XL-DKC due to Dyskerin deficiency
DNMT3B	Immunodeficiency with centromeric instability and facial anomalies (ICF1)
DOCK2	DOCK2 deficiency
DOCK8	DOCK8 deficiency
ELANE	Elastase deficiency (SCN1), cyclic neutropenia
EPG5	Vici syndrome due to EPG5 deficiency
FADD	FADD deficiency
FAS	ALPS-FAS
FASLG	ALPS-FASLG
FERMT3	Leukocyte adhesion deficiency type 3
FOXN1	Winged helix deficiency (nude) AAB: syndromic SCID

FOXP3	IPEX, immune dysregulation, polyendocrinopathy, enteropathy X-linked
FPR1	Localized juvenile periodontitis
G6PC3	G6PC3 deficiency (SCN4)
GATA2	GATA2 deficiency
GFI1	GFI 1 deficiency (SCN2)
HAX1	Kostmann Disease (SCN3)
ICOS	ICOS deficiency
IFIH1	Aicardi-Goutieres syndrome 7
IFNGR1	IFN-γ receptor 1 deficiency
IFNGR2	IFN-γ receptor 2 deficiency
IGLL1	λ5 deficiency
IKBKB	IKBKB deficiency
IL10	IL-10 deficiency
IL10RA	IL-10Rα deficiency
IL10RB	IL-10Rβ deficiency
IL12B	IL-12p40 deficiency
IL12RB1	IL-12 and IL-23 receptor β1 chain deficiency
IL17F	IL-17F deficiency
IL17RA	IL-17RA deficiency
IL17RC	IL-17RC deficiency
IL1RN	IL1RN
IL21	IL-21 deficiency
IL21R	IL-21R deficiency
IL2RA	CD25 deficiency
IL2RG	γc deficiency
IL36RN	DITRA – Deficiency of IL-36 receptor antagonist
IL7R	IL7Rα deficiency
IRAK4	IRAK-4 deficiency

IRF7	IRF7 deficiency
IRF8	IRF8 deficiency
ISG15	ISG15 deficiency
ITCH	ITCH deficiency
ITGB2	Leukocyte adhesion deficiency type 1
ITK	lymphoproliferative syndrome type 1 (LPFS1)
JAGN1	JAGN1 deficiency
JAK3	JAK3 deficiency
LAMTOR2	P14/LAMTOR2 deficiency
LCK	LCK deficiency
LIG4	DNA ligase IV deficiency
LPIN2	Chronic recurrent multifocal osteomyelitis and congenital dyserythropoietic anemia (Majeed syndrome)
LRBA	LRBA deficiency
LYST	Chediak-Higashi syndrome
MAGT1	X-linked immunodeficiency with magnesium defect, Epstein-Barr virus infection, and neoplasia (XMEN)
MALT1	MALT1 deficiency
MAP3K14	NIK deficiency
MEFV	Familial Mediterranean Fever
MOGS	MOGS deficiency
MVK	Mevalonate kinase deficiency
MYD88	MyD88 deficiency
NBN	Nijmegen breakage syndrome
NCF2	Autosomal recessive CGD
NCF4	Autosomal recessive CGD
NFAT5	NFAT5 haploinsufficiency
NFKB2	NFKB2 deficiency

NFKBIA	Anhidrotic ectodermal dysplasia with T-cell immunodeficiency (EDA-ID), IKBA gain
NHEJ1	Cernunnos/XLF deficiency
NHP2	AR-DKC due to nucleolar protein family A member 2 (NHP2) deficiency
NLRC4	NLRC4-MAS (macrophage activating syndrome), Familial cold autoinflammatory syndrome 4
NLRP12	Familial cold autoinflammatory syndrome 2
NLRP3	Muckle-Wells syndrome, Familial cold autoinflammatory syndrome 1, Neonatal onset multisystem inflammatory disease (NOMID) or chronic infantile neurologic cutaneous and articular syndrome (CINCA)
NOD2	Blau syndrome
NOP10	AR-DKC due to nucleolar protein family A member 3 (NHP3) or NOP10 deficiency
ORAI1	ORAI-I deficiency
PARN	AR-DKC due to PARN deficiency
PGM3	PGM3 deficiency
PIK3CD	Activated PI3K-δ
PIK3R1	PI3KR1 deficiency, PI3KR1 loss of function
PLCG2	PLAID (PLCγ2 associated antibody deficiency and immune dysregulation), Familial cold autoinflammatory syndrome 3, APLAID (autoinflammation and PLCγ2 associated antibody deficiency and immune dysregulation)
PMM2	PMM2-congenital disorder of glycosylation (CDG-Ia)
PNP	Purine nucleoside phosphorylase (PNP) deficiency
POLE	FILS syndrome
PRF1	Perforin deficiency (FHL2)
PRKCD	PRKC delta deficiency
PRKDC	DNA PKcs deficiency
PSMB8	CANDLE (chronic atypical neutrophilic dermatitis with lipodystrophy)
PSTPIP1	Pyogenic sterile arthritis, pyoderma gangrenosum, acne (PAPA) syndrome
PTPRC	CD45 deficiency
RAB27A	Griscelli syndrome, type 2
RAC2	Rac 2 deficiency
RAG1	RAG 1 deficiency

RAG2	RAG 2 deficiency
RBCK1	Polyglucosan body myopathy, early-onset, with or without immunodeficiency (PBMEI)
RFX5	MHC class II deficiency group C
RFXANK	MHC class II deficiency group B
RFXAP	MHC class II deficiency group D
RHOH	RhoH deficiency
RMRP	Cartilage hair hypoplasia
RNASEH2A	RNASEH2A deficiency, Aicardi-Goutieres syndrome 4
RNASEH2B	RNASEH2B deficiency, Aicardi-Goutieres syndrome 2
RNASEH2C	RNASEH2C deficiency, Aicardi-Goutieres syndrome 3
RORC	RORc deficiency
RTEL1	AR-DKC due to regulator of telomere elongation (RTEL1) deficiency
SAMHD1	SAMHD1 deficiency, Aicardi-Goutieres syndrome 5
SEMA3E	CHARGE syndrome
SH2D1A	SH2D1A deficiency (XLP1)
SH3BP2	Cherubism
SLC29A3	SLC29A3 mutation
SLC35C1	Leukocyte adhesion deficiency type 2
SLC37A4	Glycogen storage disease type 1b
SLC7A7	lysinuric protein intolerance
SMARCAL1	Schimke Immunoosseous Dysplasia
SP110	Hepatic veno-occlusive disease with immunodeficiency
SPINK5	Comel-Netherton syndrome
STAT1	STAT1 deficiency
STAT2	STAT2 deficiency
STAT3	AD-HIES (Job or Buckley Syndrome), STAT3 GOF mutations
STAT5B	STAT5b deficiency

STIM1	STIM1 deficiency
STK4	MST1 deficiency
STX11	Syntaxin 11 deficiency, (FHL4)
STXBP2	STXBP2 / Munc18-2 deficiency (FHL5)
TAP1	MHC class I deficiency
TAP2	MHC class I deficiency
TAPBP	MHC class I deficiency
TBX1	DiGeorge syndrome
TAZ	Barth syndrome
TBX1	DiGeorge syndrome
TCN2	Transcobalamin 2 deficiency
TERC	AD-DKC due to TERC deficiency
TERT	AD-DKC due to TERT deficiency
TICAM1	TRIF deficiency
TINF2	AD-DKC due to TINF2 deficiency
TLR3	TLR3 deficiency
TMC6	EVER1 deficiency
TMC8	EVER2 deficiency
TMEM173	STING–associated vasculopathy, infantile onset
TNFRSF13B	TACI deficiency
TNFRSF13C	BAFF receptor deficiency
TNFRSF1A	TNF receptor-associated periodic syndrome (TRAPS)
TNFRSF4	OX40 deficiency
TNFSF12	TWEAK deficiency
TPP2	Tripeptidyl-Peptidase II Deficiency
TRAF3	TRAF3 deficiency
TRAF3IP2	ACT1 deficiency
TREX1	TREX1 deficiency, Aicardi-Goutieres syndrome

TRNT1	TRNT1 deficiency
TTC7A	Immunodeficiency with multiple intestinal atresias
TYK2	Tyk2 deficiency
UNC13D	UNC13D / Munc13-4 deficiency (FHL3)
UNC93B1	UNC93B1 deficiency
UNG	UNG deficiency
VPS13B	Cohen syndrome
VPS45	VPS45 deficiency (SCN5)
WAS	Wiskott-Aldrich syndrome, X-linked neutropenia/ myelodysplasia
WIPF1	WIP deficiency
XIAP	XIAP deficiency (XLP2)
ZAP70	ZAP-70 deficiency
ZBTB24	Immunodeficiency with centromeric instability and facial anomalies (ICF2)

Supplementary Table 3. Genes on the 407 gene PI panel and their associated conditions

Gene	Condition(s)
ACD	autosomal dominant and autosomal recessive dyskeratosis congenita (DC) spectrum disorders
ACP5	autosomal recessive spondyloenchondrodysplasia with immune dysregulation (SED)
ACTB	autosomal dominant Baraitser-Winter cerebrofrontofacial (BWCFF) syndrome
ADA	autosomal recessive severe combined immunodeficiency due to adenosine deaminase deficiency
ADA2	autosomal recessive deficiency of adenosine deaminase 2 (DADA2)
ADAM17	autosomal recessive inflammatory skin and bowel disease (ISBD)
ADAR	autosomal dominant dyschromatosis symmetrica hereditaria (DSH)
AICDA	autosomal recessive, and rarely, autosomal dominant, hyper-IgM syndrome (HIGM)
AIRE	autosomal recessive and autosomal dominant autoimmune polyendocrinopathy with candidiasis and ectodermal dysplasia (APECED)
AK2	autosomal recessive reticular dysgenesis
ALG6	autosomal recessive ALG6-congenital disorder of glycosylation (CDG-Ic)
ANGPT1	autosomal dominant hereditary angioedema

ANKZF1	infantile onset inflammatory bowel disease
AP3B1	autosomal recessive Hermansky-Pudlak syndrome (HPS) type 2
AP3D1	autosomal recessive Hermansky-Pudlak syndrome
ARHGEF1	autosomal recessive ARHGEF1-related primary antibody deficiency
ARPC1B	autosomal recessive ARPC1B deficiency
ASAH1	autosomal recessive acid ceramidase deficiency, also known as Farber lipogranulomatosis or Farber disease
ATM	autosomal recessive ataxia-telangiectasia (A-T) and autosomal dominant predisposition to breast, pancreatic and possibly prostate cancer
ATP6AP1	X-linked recessive ATP6AP1 deficiency
B2M	autosomal recessive hereditary major histocompatibility complex (MHC) class I deficiency
BACH2	autosomal dominant BACH2 deficiency
BCL10	combined immunodeficiency
BCL11B	autosomal dominant BCL11B deficiency
BLM	autosomal recessive Bloom syndrome
BLNK	autosomal recessive BLNK deficiency
BLOC1S3	autosomal recessive Hermansky-Pudlak syndrome
BLOC1S6	autosomal recessive Hermansky-Pudlak syndrome (HPS) type 9
BTK	X-linked recessive agammaglobulinemia (XLA)
C17orf62	autosomal recessive chronic granulomatous disease
C1QA	autosomal recessive C1q deficiency
C1QB	autosomal recessive C1q deficiency
C1QC	autosomal recessive C1q deficiency
C1S	autosomal recessive C1s deficiency
C2	autosomal recessive complement component 2 (C2) deficiency
C3	autosomal recessive C3 deficiency
C5	autosomal recessive complement component 5 (C5) deficiency
C6	autosomal recessive complement component 6 (C6) deficiency
C7	autosomal recessive complement component 7 (C7) deficiency
C8A	autosomal recessive C8 alpha deficiency
C8B	autosomal recessive C8 beta deficiency
C9	autosomal recessive complement component 9 (C9) deficiency

CARD11	autosomal recessive combined immunodeficiency due to CARD11 deficiency
CARD14	autosomal dominant CAMPS (CARD14-mediated psoriasis)
CARD8	autosomal dominant Crohn's disease
CARD9	autosomal recessive CARD9 deficiency
CARMIL2	autosomal recessive RLTPR (CARMIL2) deficiency
CASP10	autosomal dominant and autosomal recessive autoimmune lymphoproliferative syndrome (ALPS-CASP10)
CASP8	autosomal recessive caspase-8 deficiency state (CEDS)
CBL	autosomal dominant Noonan-like syndrome with or without juvenile myelomonocytic leukemia
CCBE1	autosomal recessive Hennekam lymphangiectasia-lymphedema syndrome
CD19	autosomal recessive common variable immune deficiency (CVID) due to CD19 deficiency
CD247	autosomal recessive severe combined immunodeficiency due to CD3-zeta deficiency
CD27	autosomal recessive CD27 deficiency
CD3D	autosomal recessive severe combined immunodeficiency due to CD3-delta deficiency
CD3E	autosomal recessive severe combined immunodeficiency due to CD3-epsilon deficiency
CD3G	autosomal recessive combined immunodeficiency due to CD3-gamma deficiency
CD40	autosomal recessive hyper IgM syndrome (HIGM)
CD40LG	X-linked hyper-IgM syndrome (HIGM)
CD46	autosomal dominant atypical hemolytic uremic syndrome (aHUS)
CD55	autosomal recessive complement hyperactivation, angiopathic thrombosis, and protein-losing enteropathy syndrome
CD59	autosomal recessive CD59-mediated hemolytic anemia, with or without immune-mediated polyneuropathy (HACD59)
CD79A	autosomal recessive agammaglobulinemia due to Igα deficiency
CD79B	autosomal recessive agammaglobulinemia due to Igβ deficiency
CD81	autosomal recessive common variable immunodeficiency due to CD81 deficiency
CD8A	autosomal recessive familial CD8 deficiency
CDC42	autosomal dominant Takenouchi-Kosaki syndrome
CDCA7	autosomal recessive immunodeficiency-centromeric instability-facial anomalies (ICF) syndrome
CEBPE	autosomal recessive neutrophil-specific granule deficiency
CFB	autosomal dominant atypical hemolytic uremic syndrome

CFD	autosomal recessive complement factor D deficiency (CFDD)
CFH	autosomal dominant atypical hemolytic uremic syndrome
CFI	autosomal recessive complement factor I deficiency
CFP	X-linked recessive properdin deficiency
CHD7	autosomal dominant CHARGE syndrome
CIB1	autosomal recessive epidermodysplasia verruciformis due to CIB1 deficiency
CIITA	autosomal recessive major histocompatibility complex (MHC) class II deficiency
CLCN7	autosomal recessive osteopetrosis
CLPB	autosomal recessive 3-methylglutaconic aciduria with cataracts, neurologic involvement, and neutropenia (MEGCANN)
COL7A1	autosomal dominant dystrophic epidermolysis bullosa (DDEB)
COPA	autosomal dominant autoimmune interstitial lung, joint, and kidney disease (AILJK)
CORO1A	autosomal recessive severe combined immunodeficiency due to coronin-1A deficiency
CR2	autosomal recessive hypogammaglobulinemia due to CD21 deficiency
CSF2RA	X-linked primary pulmonary alveolar proteinosis (PAP)
CSF2RB	autosomal recessive pulmonary alveolar proteinosis (PAP)
CSF3R	autosomal dominant hereditary neutrophilia
CTC1	autosomal recessive cerebroretinal microangiopathy with calcifications and cysts type 1 (CRMCC1), also known as Coats plus syndrome
CTLA4	autosomal dominant CTLA4 haploinsufficiency
CTPS1	autosomal recessive combined immunodeficiency due to CTPS1 deficiency
CTSC	autosomal recessive Papillon-Lefevre syndrome
CXCR2	autosomal recessive myelokathexis
CXCR4	autosomal dominant warts, hypogammaglobulinemia, infections, and myelokathexis syndrome (WHIMS)
CYBA	autosomal recessive chronic granulomatous disease (CGD)
CYBB	X-linked recessive chronic granulomatous disease (CGD)
CYP27A1	autosomal recessive cerebrotendinous xanthomatosis (CTX)
DCLRE1C	autosomal recessive severe combined immunodeficiency due to DCLRE1C (Artemis) deficiency
DDX58	autosomal dominant Singleton-Merten syndrome
DEF6	DEF6 deficiency
DGAT1	autosomal recessive congenital chronic diarrhea
DIAPH1	autosomal dominant deafness with or without thrombocytopenia (DFNA1)

DKC1	X-linked dyskeratosis congenita spectrum disorders (DC)
DNAJC21	autosomal recessive Shwachman-Diamond syndrome due to DNAJC21 deficiency
DNASE1L3	autosomal recessive systemic lupus erythematosus
DNASE2	autosomal recessive DNase II deficiency
DNMT3B	autosomal recessive immunodeficiency, centromeric instability, and facial anomalies (ICF) syndrome
DOCK2	autosomal recessive combined immunodeficiency (CID) due to DOCK2 deficiency
DOCK8	autosomal recessive DOCK8 deficiency
DSG1	autosomal dominant keratosis palmoplantaris striata
DTNBP1	autosomal recessive Hermansky-Pudlak syndrome
DUOX2	autosomal dominant partial iodide organification defect (PIOD) and autosomal recessive thyroid [illegible]
EFL1	autosomal recessive Shwachman-Diamond syndrome
EIF2AK3	autosomal recessive Wolcott-Rallison syndrome (WRS)
ELANE	autosomal dominant ELANE-related neutropenia, including both congenital
EPG5	autosomal recessive Vici syndrome
ERBIN	autosomal dominant elevated IgE, eosinophilic esophagitis, joint hypermobility & vascular anomalies
ERCC2	autosomal recessive photosensitive trichothiodystrophy (TTD)
ERCC3	autosomal recessive xeroderma pigmentosum/Cockayne syndrome
ERCC6L2	autosomal recessive ERCC6L2 (Hebo) deficiency
EXTL3	autosomal recessive EXTL3 deficiency
FADD	autosomal recessive FADD deficiency
FANCA	autosomal recessive Fanconi anemia type A (FA-A)
FANCB	X-linked Fanconi anemia type B (FA-B)
FANCE	autosomal recessive Fanconi anemia, type E (FA-E)
FANCF	autosomal recessive Fanconi anemia, type F (FA-F)
FANCI	autosomal recessive Fanconi anemia, type I (FA-I)
FANCL	autosomal recessive Fanconi anemia, type L (FA-L)
FAS	autosomal dominant autoimmune lymphoproliferative syndrome (ALPS-FAS)
FASLG	autosomal recessive autoimmune lymphoproliferative syndrome (ALPS-FASLG)
FAT4	autosomal recessive Hennekam lymphangiectasia-lymphedema syndrome
FCHO1	autosomal recessive combined immunodeficiency due to FCHO1 deficiency

FERMT1	autosomal recessive Kindler syndrome
FERMT3	autosomal recessive leukocyte adhesion deficiency, type 3 (LAD3)
FOXI3	FOXI3 deficiency
FOXN1	autosomal recessive severe combined immunodeficiency due to FOXN1 deficiency
FOXP3	X-linked recessive immunodysregulation, polyendocrinopathy, and enteropathy (IPEX syndrome)
FPR1	autosomal dominant periodontitis
G6PC	autosomal recessive glycogen storage disease type Ia (GSDIa)
G6PC3	autosomal recessive severe congenital neutropenia
G6PD	X-linked glucose-6-phosphate dehydrogenase deficiency
GATA2	autosomal dominant GATA2 deficiency
GFI1	autosomal dominant severe congenital neutropenia due to GFI1 deficiency
GINS1	autosomal recessive immunodeficiency due to GINS1 deficiency
GTF2E2	autosomal recessive trichothiodystrophy
GTF2H5	autosomal recessive trichothiodystrophy (TTD)
GUCY2C	autosomal dominant congenital sodium diarrhea
HAX1	autosomal recessive severe congenital neutropenia due to HAX1 deficiency
HELLS	autosomal recessive immunodeficiency-centromeric instability-facial anomalies (ICF) syndrome
HMOX1	autosomal recessive heme oxygenase 1 deficiency (HMOX1D)
HPS1	autosomal recessive Hermansky-Pudlak syndrome 1 (HPS1)
HPS3	autosomal recessive Hermansky-Pudlak syndrome 3 (HPS3)
HPS4	autosomal recessive Hermansky-Pudlak syndrome
HPS5	autosomal recessive Hermansky-Pudlak syndrome
HPS6	autosomal recessive Hermansky-Pudlak syndrome
HTRA2	autosomal recessive 3-methylglutaconic aciduria
HYOU1	autosomal recessive HYOU1-related immunodeficiency and hypoglycemia
ICOS	autosomal recessive common variable immunodeficiency (CVID) due to ICOS deficiency
ICOSLG	autosomal recessive combined immunodeficiency
IFIH1	autosomal dominant Aicardi-Goutieres syndrome (AGS)
IFNAR1	susceptibility to infections due to IFNAR1 deficiency and Evans syndrome
IFNAR2	autosomal recessive IFNAR2 deficiency

IFNGR1	autosomal recessive Mendelian susceptibility to mycobacterial disease (MSMD) due to interferon-gamma receptor 1 deficiency.
IFNGR2	autosomal recessive Mendelian susceptibility to mycobacterial disease (MSMD)
IGLL1	autosomal recessive agammaglobulinemia
IKBKB	autosomal dominant anhidrotic ectodermal dysplasia with immunodeficiency (EDA-ID) due to IKBKB gain-of-function
IL10	autosomal recessive infantile onset inflammatory bowel disease (IBD) due to IL-10 deficiency
IL10RA	autosomal recessive early onset inflammatory bowel disease (IBD), due to interleukin 10 receptor alpha deficiency
IL10RB	autosomal recessive inflammatory bowel disease (IBD) due to interleukin 10 receptor beta deficiency
IL12B	autosomal recessive Mendelian susceptibility to mycobacterial disease due to interleukin 12 deficiency
IL12RB1	autosomal recessive Mendelian susceptibility to mycobacterial disease (MSMD)
IL12RB2	autosomal recessive IL12RB2 deficiency
IL17F	autosomal dominant chronic mucocutaneous candidiasis
IL17RA	autosomal recessive chronic mucocutaneous candidiasis (CMC) due to IL17RA deficiency
IL17RC	autosomal recessive chronic mucocutaneous candidiasis
IL1RN	autosomal recessive interleukin 1 receptor antagonist deficiency (DIRA)
IL21	autosomal recessive early onset inflammatory bowel disease and common variable immunodeficiency (CVID)
IL21R	autosomal recessive combined immunodeficiency due to interleukin 21 receptor deficiency
IL23R	autosomal recessive IL23R deficiency
IL2RA	autosomal recessive CD25 deficiency
IL2RB	autosomal recessive immunodeficiency with lymphoproliferation and autoimmunity due to CD122 deficiency
IL2RG	X-linked recessive severe combined immunodeficiency
IL36RN	autosomal recessive deficiency of interleukin-36 receptor antagonist (DITRA)
IL6R	IL6 receptor deficiency
IL6ST	autosomal dominant hyper IgE syndrome (HIES) and autosomal recessive Stuve-Wiedemann syndrome (SWS)
IL7R	autosomal recessive severe combined immunodeficiency due to IL7R-alpha deficiency
IRAK4	autosomal recessive IRAK-4 deficiency
IRF2BP2	autosomal dominant common variable immunodeficiency due to IRF2BP2 deficiency

IRF4	autosomal recessive IRF4 deficiency
IRF7	autosomal recessive severe influenza disease
IRF8	autosomal dominant susceptibility to mycobacterial disease (MSMD) and autosomal recessive natural killer (NK) cell deficiency
IRF9	autosomal recessive IRF9 deficiency
ISG15	autosomal recessive Mendelian susceptibility to mycobacterial disease (MSMD)
ITCH	autosomal recessive ITCH deficiency
ITGAM	systemic lupus erythematous
ITGB2	autosomal recessive leukocyte adhesion deficiency type 1 (LAD1)
ITK	autosomal recessive ITK deficiency
JAGN1	autosomal recessive severe congenital neutropenia due to JAGN1 deficiency
JAK1	autosomal dominant immune dysregulation and hypereosinophilic syndrome and autosomal recessive Mendelian susceptibility to mycobacterial disease
JAK3	autosomal recessive severe combined immunodeficiency due to JAK3 deficiency
KDM6A	X-linked dominant Kabuki syndrome
KMT2A	autosomal dominant Wiedemann-Steiner syndrome (WDSTS)
KMT2D	autosomal dominant Kabuki syndrome
LAMTOR2	autosomal recessive LAMTOR2 deficiency
LAT	autosomal recessive combined immunodeficiency due to LAT deficiency
LCK	autosomal recessive combined immunodeficiency due to LCK deficiency
LCT	autosomal recessive congenital lactase deficiency
LIG1	autosomal recessive ligase I deficiency
LIG4	autosomal recessive LIG4 syndrome
LIPA	autosomal recessive lysosomal acid lipase (LAL) deficiency
LPIN2	autosomal recessive Majeed syndrome
LRBA	autosomal recessive LRBA deficiency
LRRC8A	autosomal dominant agammaglobulinemia due to LRRC8A deficiency
LYN	autoinflammatory disease
LYST	autosomal recessive Chediak-Higashi syndrome (CHS)
MAGT1	X-linked recessive immunodeficiency with magnesium defect, Epstein-Barr virus infection, and neoplasia (XMEN)
MALT1	autosomal recessive combined immunodeficiency due to MALT1 deficiency

MAP3K14	autosomal recessive combined immunodeficiency due to NF-κB-inducing kinase (NIK) deficiency
MCM4	autosomal recessive MCM4 deficiency
MEFV	autosomal recessive familial Mediterranean fever (FMF)
MKL1	autosomal recessive MKL1 deficiency
MOGS	autosomal recessive MOGS-congenital disorder of glycosylation (CDG-IIb)
MPLKIP	autosomal recessive non-photosensitive trichothiodystrophy
MS4A1	autosomal recessive CD20 deficiency
MSN	X-linked moesin deficiency
MTHFD1	autosomal recessive methylenetetrahydrofolate dehydrogenase 1 deficiency
MVK	autosomal recessive mevalonate kinase deficiency
MYD88	autosomal recessive MyD88 deficiency
MYO5B	autosomal recessive microvillus inclusion disease (MVID)
MYSM1	autosomal recessive MYSM1 deficiency
NBAS	autosomal recessive infantile liver failure
NBN	autosomal recessive Nijmegen breakage syndrome (NBS)
NCF2	autosomal recessive chronic granulomatous disease (CGD)
NCF4	autosomal recessive chronic granulomatous disease (CGD)
NCSTN	autosomal dominant hidradenitis suppurativa (HS) due to NCSTN deficiency
NEUROG3	autosomal recessive congenital malabsorptive diarrhea
NFAT5	autosomal dominant immunodeficiency and autoimmune enterocolopathy
NFE2L2	autosomal dominant immunodeficiency, developmental delay, and hypohomocysteinemia due to NFE2L2 gain-of-function
NFKB1	autosomal dominant common variable immunodeficiency due to NKFB1 deficiency
NFKB2	autosomal dominant deficient anterior pituitary with variable immune deficiency (DAVID) syndrome
NFKBIA	autosomal dominant anhidrotic ectodermal dysplasia with T-cell immunodeficiency (EDA-ID)
NHEJ1	autosomal recessive severe combined immunodeficiency due to Cernunnos/XLF deficiency
NHP2	autosomal recessive NHP2-related dyskeratosis congenita (DC) spectrum disorders
NLRC4	autosomal dominant familial cold autoinflammatory syndrome (FCAS)
NLRP1	autosomal dominant NLRP1 gain of function (GOF) syndrome
NLRP12	autosomal dominant familial cold autoinflammatory syndrome (FCAS)

NLRP3	autosomal dominant cryopyrin-associated periodic syndrome (CAPS)
NOD2	autosomal dominant Blau syndrome
NOP10	NOP10-related dyskeratosis congenita (DC) spectrum disorders
NSMCE3	autosomal recessive NSMCE3 deficiency
OAS1	autosomal dominant pulmonary alveolar proteinosis with hypogammaglobulinemia
ORAI1	autosomal dominant tubular aggregate myopathy 2 (TAM2)
OSTM1	autosomal recessive OSTM1 deficiency associated osteopetrosis
OTULIN	autosomal recessive otulipenia/ORAS
PARN	autosomal recessive dyskeratosis congenita
PAX1	autosomal recessive otofaciocervical syndrome
PEPD	autosomal recessive prolidase deficiency (PD)
PGM3	autosomal recessive PGM3-congenital disorder of glycosylation (CDG)
PIK3CD	autosomal dominant activated phosphoinositide 3-kinase δ (PI3K-delta) syndrome
PIK3R1	autosomal dominant SHORT syndrome
PLCG2	autosomal dominant familial cold autoinflammatory syndrome
PMM2	autosomal recessive PMM2-congenital disorder of glycosylation (CDG-Ia)
PNP	autosomal recessive purine nucleoside phosphorylase deficiency
POLA1	X-linked pigmentary reticulate disorder
POLD1	autosomal dominant predisposition to colonic adenomatous polyps and colon cancer
POLE	an autosomal dominant predisposition to colonic adenomatous polyps and colon cancer
POLE2	combined immunodeficiency
POLR3A	autosomal recessive hypomyelinating leukodystrophy 7
POMP	autosomal dominant proteasome-associated autoinflammatory syndrome type 2 (PRAAS2)
PRF1	autosomal recessive familial hemophagocytic lymphohistiocytosis type 2 (FHL2)
PRKCD	autosomal recessive PRKC delta deficiency
PRKDC	autosomal recessive severe combined immunodeficiency due to DNA PKcs deficiency
PSENEN	autosomal dominant hidradenitis suppurativa (HS) due to PSENEN deficiency
PSMA3	proteasome-associated autoinflammatory syndrome-1
PSMB4	autosomal recessive chronic atypical neutrophilic dermatosis with lipodystrophy and elevated temperature (CANDLE) syndrome
PSMB8	autosomal recessive proteasome-associated autoinflammatory syndrome (PRAAS)

PSMG2	autosomal recessive chronic atypical neutrophilic dermatosis with lipodystrophy and elevated temperature (CANDLE) syndrome
PSTPIP1	autosomal dominant pyogenic sterile arthritis, pyoderma gangrenosum, and acne (PAPA) syndrome
PTPRC	autosomal recessive severe combined immunodeficiency due to CD45 deficiency
RAB27A	autosomal recessive Griscelli syndrome type 2 (GS2)
RAC2	autosomal dominant neutrophil immunodeficiency syndrome
RAG1	autosomal recessive severe combined immunodeficiency due to RAG1 deficiency
RAG2	autosomal recessive severe combined immunodeficiency due to RAG2 deficiency
RANBP2	autosomal dominant infection-induced acute necrotizing encephalopathy
RASGRP1	autosomal recessive RASGRP1 deficiency
RBCK1	autosomal recessive polyglucosan body myopathy with or without immunodeficiency (PGBM1)
RELA	autosomal dominant chronic mucocutaneous ulceration
RELB	autosomal recessive combined immunodeficiency due to RELB deficiency
RFX5	autosomal recessive hereditary major histocompatibility complex (MHC) class II deficiency
RFXANK	autosomal recessive hereditary major histocompatibility complex (MHC) class II deficiency
RFXAP	autosomal recessive hereditary major histocompatibility complex (MHC) class II deficiency
RHOH	autosomal recessive T-cell immunodeficiency with epidermodysplasia verruciformis
RIPK1	autosomal recessive RIPK1 deficiency
RMRP	autosomal recessive cartilage-hair hypoplasia-anauxetic dysplasia (CHH-AD) spectrum disorders
RNASEH2A	autosomal recessive Aicardi Goutieres syndrome 4 (AGS4)
RNASEH2B	autosomal recessive Aicardi Goutieres syndrome 2 (AGS2)
RNASEH2C	autosomal recessive Aicardi Goutieres syndrome 3 (AGS3)
RNF113A	X-linked trichothiodystrophy
RNF168	autosomal recessive radiosensitivity, immunodeficiency, dysmorphic features, and learning difficulties (RIDDLE) syndrome
RNF31	autosomal recessive autoinflammation, immunodeficiency, amyelopectinosis, and lymphangiectasia
RNU4ATAC	autosomal recessive Roifman syndrome and Lowry-Wood syndrome
RORC	autosomal recessive RORC deficiency
RPSA	autosomal dominant isolated congenital asplenia (ICA) due to RPSA deficiency
RTEL1	autosomal dominant and autosomal recessive dyskeratosis congenita (DC) spectrum disorders

SAMD9	autosomal dominant myelodysplasia, infection, restriction of growth, adrenal hypoplasia and insufficiency, genital abnormalities, and enteropathy (MIRAGE) syndrome
SAMD9L	autosomal dominant ataxia-pancytopenia (AP) syndrome
SAMHD1	autosomal recessive Aicardi-Goutieres syndrome 5 (AGS5)
SAR1B	autosomal recessive chylomicron retention disease (CMRD)
SCO2	autosomal recessive cardioencephalomyopathy due to mitochondrial complex IV deficiency
SEC61A1	autosomal dominant tubulointerstitial kidney disease
SEMA3E	chronic kidney disease, seizures and hypothyroidism
SERPING1	autosomal dominant hereditary angioedema types I & II
SH2D1A	X-linked recessive lymphoproliferative syndrome 1 (XLP1)
SH3BP2	autosomal dominant cherubism
SH3KBP1	X-linked recessive antibody deficiency
SI	autosomal recessive sucrase-isomaltase deficiency
SIAE	susceptibility to juvenile idiopathic arthritis
SKIV2L	autosomal recessive trichohepatoenteric syndrome
SLC26A3	autosomal recessive congenital secretory chloride diarrhea
SLC29A3	autosomal recessive histiocytosis-lymphadenopathy plus syndrome
SLC35C1	autosomal recessive SLC35C1-congenital disorder of glycosylation (CDG-IIc)
SLC37A4	autosomal recessive glycogen storage disease type Ib (GSD Ib)
SLC39A7	autosomal recessive agammaglobulinemia due to SLC39A7 deficiency
SLC46A1	autosomal recessive hereditary folate malabsorption
SLC5A1	autosomal recessive glucose-galactose malabsorption (GGM)
SLC7A7	autosomal recessive lysinuric protein intolerance (LPI)
SLC9A3	autosomal recessive congenital sodium diarrhea
SLX4	autosomal recessive Fanconi anemia, type P (FA-P)
SMARCAL1	autosomal recessive Schimke immunoosseous dysplasia (SIOD)
SMARCD2	autosomal recessive neutrophil-specific granule deficiency
SNX10	autosomal recessive osteopetrosis
SP110	autosomal recessive hepatic venoocclusive disease with immunodeficiency (VODI)
SPINK5	autosomal recessive Netherton syndrome
SPINT2	autosomal recessive congenital sodium diarrhea

SPPL2A	autosomal recessive mendelian susceptibility to mycobacterial disease
SRP54	autosomal dominant Shwachman-Diamond syndrome due to SRP54 deficiency
SRP72	autosomal dominant familial bone marrow failure
STAT1	autosomal recessive STAT1 deficiency and autosomal dominant STAT1 gain-of-function associated chronic mucocutaneous candidiasis
STAT2	autosomal recessive STAT2 deficiency
STAT3	autosomal dominant Hyper-IgE syndrome
STAT4	paracoccidiomycosis
STAT5B	autosomal dominant STAT5B deficiency
STIM1	autosomal dominant tubular aggregate myopathy 1 (TAM1), and autosomal recessive STIM1 deficiency
STK4	autosomal recessive combined immunodeficiency due to MST1 deficiency
STN1	autosomal recessive cerebroretinal microangiopathy with calcifications and cysts (CRMCC)
STX11	autosomal recessive familial hemophagocytic lymphohistiocytosis type 4 (FHL4)
STX3	autosomal recessive microvillus inclusion disease (MVID)
STXBP2	autosomal recessive familial hemophagocytic lymphohistiocytosis type 5 (FHL5)
TAOK2	autosomal recessive primary immunodeficiency
TAP1	autosomal recessive hereditary major histocompatibility complex (MHC) class I deficiency
TAP2	autosomal recessive hereditary major histocompatibility complex (MHC) class I deficiency
TAPBP	autosomal recessive hereditary major histocompatibility complex (MHC) class I deficiency
TAZ	X-linked recessive Barth Syndrome (BTHS), also known as 3-methylglutaconic aciduria type II
TBX1	autosomal dominant DiGeorge/velocardiofacial syndrome
TCF3	autosomal dominant agammaglobulinemia due to E47 transcription factor deficiency
TCIRG1	autosomal recessive osteopetrosis due to TCIRG1 deficiency
TCN2	autosomal recessive transcobalamin II deficiency
TERC	autosomal dominant TERC-related dyskeratosis congenita (DC) spectrum disorders
TERT	both autosomal dominant and autosomal recessive TERT-related dyskeratosis congenita (DC) spectrum disorders
TFRC	autosomal recessive combined immunodeficiency due to TFRC deficiency
TGFB1	autosomal dominant Camurati-Engelmann disease (CED) and autosomal dominant common variable immunodeficiency
TGFBR1	autosomal dominant nonsyndromic thoracic aortic aneurysms and aortic dissections (TAAD)

TGFBR2	autosomal dominant Loeys-Dietz syndrome 2 (LDS2)
THBD	autosomal dominant thrombomodulin-associated coagulopathy (TM-AC)
TICAM1	autosomal recessive susceptibility to herpes simplex encephalitis (HSE)
TIMM50	autosomal recessive 3-methylglutaconic aciduria
TINF2	autosomal dominant TINF2-related dyskeratosis congenita (DC) spectrum disorders
TLR3	susceptibility to herpes simplex encephalitis
TMC6	autosomal recessive epidermodysplasia verruciformis (EV)
TMC8	autosomal recessive epidermodysplasia verruciformis (EV)
TMEM173	autosomal dominant infantile-onset STING-associated vasculopathy (SAVI)
TNFAIP3	autosomal dominant familial Behcet-like autoinflammatory syndrome
TNFRSF11A	autosomal dominant hereditary recurrent fevers
TNFRSF13B	autosomal recessive common variable immunodeficiency (CVID) due to TACI deficiency
TNFRSF13C	autosomal recessive common variable immunodeficiency
TNFRSF1A	autosomal dominant tumor necrosis factor receptor-associated periodic syndrome (TRAPS)
TNFRSF4	autosomal recessive combined immunodeficiency
TNFRSF6B	systemic lupus erythematosus
TNFRSF9	autosomal recessive CD137 deficiency
TNFSF11	autosomal recessive osteopetrosis
TNFSF12	autosomal dominant common variable immunodeficiency (CVID)
TONSL	autosomal recessive sponastrime dysplasia
TOP2B	autosomal dominant TOP2B deficiency
TP63	autosomal dominant acro-dermato-ungual-lacrimal-tooth (ADULT) syndrome
TPP2	autosomal recessive tripeptidyl peptidase II deficiency
TRAF3	autosomal dominant susceptibility to Evans syndrome
TRAF3IP2	autosomal recessive ACT1 deficiency
TREX1	autosomal recessive (and rarely, autosomal dominant) Aicardi-Goutieres syndrome 1 (AGS1) and autosomal dominant susceptibility to systemic lupus erythematosus (SLE)
TRNT1	autosomal recessive sideroblastic anemia with B-cell immunodeficiency, periodic fevers, and developmental delay (SIFD)
TTC37	autosomal recessive trichohepatoenteric syndrome (THES) 1
TTC7A	autosomal recessive immunodeficiency with multiple intestinal atresias

TYK2	autosomal recessive Mendelian susceptibility to mycobacterial disease
UNC13D	autosomal recessive familial hemophagocytic lymphohistiocytosis type 3 (FHL3)
UNC45A	osteo-oto-hepatoenteric syndrome
UNC93B1	autosomal recessive susceptibility to herpes simplex encephalitis
UNG	autosomal recessive Hyper IgM syndrome type 5 (HIGM5)
USB1	autosomal recessive poikiloderma with neutropenia (PN)
VAV1	autosomal dominant common variable immunodeficiency
VPS13B	autosomal recessive Cohen syndrome
VPS45	autosomal recessive severe congenital neutropenia due to VPS45 deficiency
WAS	X-linked recessive Wiskott-Aldrich syndrome and thrombocytopenia
WDR1	autosomal recessive WDR1 deficiency
WIPF1	autosomal recessive Wiskott-Aldrich syndrome due to WIP deficiency
WRAP53	autosomal recessive WRAP53-related dyskeratosis congenita (DC) spectrum disorders
XIAP	X-linked lymphoproliferative syndrome 2 (XLP2)
ZAP70	autosomal recessive combined immunodeficiency due to ZAP70 deficiency
ZBTB24	autosomal recessive immunodeficiency-centromeric instability-facial anomalies (ICF) syndrome
ZCCHC8	an autosomal dominant telomere biology disorder
ZNF341	autosomal recessive Hyper-IgE syndrome

Supplementary Figure 1. JMF Physician Questionnaire

Jeffrey Modell Centers Network

INVITAE

Jeffrey's Insights

Jeffrey Modell Foundation & Invitae

Genetic Sequencing Program Questionnaire

Physician:	Institution:	Date:

Please add as many columns as needed for patients.

	Patient 1	Patient 2	Patient 3	Patient 4	Patient 5
1. In the past 12 months, approximately how many times did this patient see a healthcare provider for symptoms of the condition?					
2. In the past 12 months, approximately how many times has this patient:					
Been admitted to the hospital?					
Visited an emergency room?					
Been admitted to the ICU?					
3. Was cost a reason this patient did not seek care for this condition? **Yes or No**					
Was cost a reason this patient did not seek genetic testing for this condition? **Yes or No**					
Was access to genetic testing an obstacle for this patient? **Yes or No**					
4. What is the annual estimated cost of care, medications, and treatment for this patient?					
5. Are the costs of care covered by the patient's insurance company or national health program? **Yes or No**					
Are the costs of genetic testing covered by the patient's insurance company or national health program? **Yes or No**					
6. Prior to genetic testing, did you have a suspicion of a particular diagnosis for this patient? **Yes or No**					
If **Yes**, did the results **confirm** or **alter** this? (Please enter **confirm** or **alter**)					
7. Based on the results of genetic testing, have you altered any of the following for this patient:					
Clinical diagnosis? **Yes or No**					
Overall disease management? **Yes or No**					
Treatment? **Yes or No**					
Genetic counseling? **Yes or No**					
8. Based on the results of genetic testing, have you identified other at-risk or affected individuals in this family? **Yes or No**					
9. Based on the results of genetic testing, have there been any changes in outcomes for this patient to date? **Yes or No**					
10. Is there an approved therapy for the diagnosis? **Yes or No**					

11. Has this patient been diagnosed with COVID-19?
Yes or No

If **Yes**, what is the current status/outcome?

12. Has this patient received treatment(s) for COVID-19?
Yes or No

If **Yes**, what treatment(s)?

**Additional Comments:**

May 2021
